# Per- and polyfluoroalkyl substances and kidney disease: Genetic associations and computational prioritization of candidate toxicogenomic pathways

**DOI:** 10.1371/journal.pcbi.1014665

**Published:** 2026-09-17

**Authors:** Dianjie Zeng, Yuxi Wang, Yinhuai Wang, Guoqiang Li, Wenpeng Wang, Zhongkun Zuo

**Affiliations:** 1 Department of Urology, The Second Xiangya Hospital at Central South University, Changsha, Hunan, China; 2 Department of Nephrology, The Second Xiangya Hospital at Central South University, Changsha, Hunan, China; 3 Xiangya School of Medicine, Central South University, Changsha, Hunan, China; 4 The Affiliated Cancer Hospital of Zhengzhou University & Henan Cancer Hospital, Zhengzhou, Henan, China; 5 Department of General Surgery, The Second Xiangya Hospital at Central South University, Changsha, Hunan, China; University of Western Ontario: Western University, CANADA

## Abstract

Per- and polyfluoroalkyl substances (PFAS) are persistent environmental pollutants with bioaccumulation potential, but their associations with kidney diseases remain incompletely understood. This study integrated Mendelian randomization and computational toxicology to examine associations between genetically predicted circulating PFAS levels and kidney disease outcomes and to prioritize candidate toxicogenomic pathway themes. Genetically predicted higher PFOA levels were inversely associated with IgA nephropathy (OR = 0.21, P = 0.004), but positively associated with hypertensive nephropathy (OR = 1.20, P < 0.001) and calculus of kidney (OR = 1.24, P = 0.016). Genetically predicted higher PFOS levels were inversely associated with IgA nephropathy (OR = 0.27, P = 0.046) and urinary tract infection (OR = 0.94, P = 0.003). A primary association was also observed between PFOA and membranous nephropathy (OR = 1.56, P = 0.028), but this association was not retained after targeted SNP-exclusion analyses and was therefore not interpreted as a robust or established causal association. Computational toxicology analyses prioritized database-derived candidate targets and pathway themes related to immune response, inflammation, oxidative stress, and apoptosis. Network-prioritized candidate nodes included CTNNB1, TP53, and EGFR in the PFOA–calculus of kidney network, IGF1 in the PFOA–hypertensive nephropathy network, and CCL2, TLR4, MMP9, and IFNG in the PFOA/PFOS–IgA nephropathy networks. In contrast, IL1B, TNF, and IL6 were observed as shared inflammatory nodes across multiple nephropathy-related networks. These candidates should be interpreted as database-derived and network-prioritized targets rather than experimentally validated causal mediators of kidney disease. Overall, this study provides a hypothesis-generating framework for exploring associations among genetically predicted PFAS-related traits, kidney disease outcomes, and candidate toxicogenomic pathway themes that require experimental validation.

## Author summary

Per- and polyfluoroalkyl substances (PFAS) are a group of persistent environmental chemicals widely detected in water, food, consumer products, and human blood. Because the kidneys are involved in filtration, tubular handling, and chemical elimination, they may be particularly relevant to long-term PFAS-related biological effects. However, it remains challenging to determine how circulating PFAS-related traits are associated with specific kidney disease outcomes and which molecular pathway themes warrant further study. In this study, we combined genetic epidemiology with computational toxicology to investigate associations between genetically predicted circulating PFAS-related traits and several kidney disease outcomes. Our genetic analyses suggested that PFOA and PFOS were associated with different kidney diseases in distinct directions. We then used computational approaches to prioritize database-derived candidate protein targets and pathway themes related to immune regulation, inflammation, oxidative stress, and apoptosis. By integrating population-level genetic evidence with computational target prioritization, this study provides a hypothesis-generating framework for future experimental studies on PFAS-related kidney health.

## 1. Introduction

Per- and polyfluoroalkyl substances (PFAS) are a class of synthetic chemicals widely used in industrial and consumer products due to their water-, oil-, and stain-resistant properties [1]. These compounds, including perfluorooctanoic acid (PFOA) and perfluorooctane sulfonate (PFOS), are persistent environmental pollutants with long half-lives in both the environment and the human body [2]. Detected in air, water, soil, and wildlife, PFAS have become ubiquitous contaminants, posing significant risks to human health [3]. The bioaccumulation potential of PFAS is of particular concern, as they persist in biological systems, leading to long-term exposure even after the discontinuation of certain PFAS-containing products [4].

The kidneys play a crucial role in maintaining homeostasis through filtration, excretion, and detoxification, making renal health essential to overall well-being. Recent epidemiological studies have suggested associations between PFAS exposure and altered renal function and kidney disease-related outcomes [5–7]. However, the biological interpretation of these associations remains poorly understood. While some studies have indicated a potential link between PFAS exposure and kidney disorders such as IgA nephropathy, hypertensive nephropathy, and calculus of kidney, the molecular processes potentially related to PFAS–kidney disease associations are yet to be clearly defined [8–10]. The complexity and multifactorial nature of kidney disease further complicate efforts to identify specific environmental factors that are associated with disease susceptibility or progression.

To address these challenges, genetic epidemiology provides a useful framework for evaluating potential causal associations between environmental exposures and disease outcomes. Mendelian randomization (MR), which uses genetic variants as instrumental variables, allows for the assessment of potential causal associations between exposures and diseases, minimizing confounding factors that often plague traditional observational studies [11,12]. It has been successfully applied to investigate the role of various environmental exposures in the development of chronic diseases, including cardiovascular disease, cancer, and metabolic disorders [13–15]. Additionally, computational toxicology methods provide an efficient means of prioritizing candidate molecular targets and pathways that may be relevant to PFAS-related toxicogenomic hypotheses. These methods leverage existing databases and computational predictions to prioritize candidate protein targets and biological pathway themes for downstream experimental follow-up.

**Fig 1:**
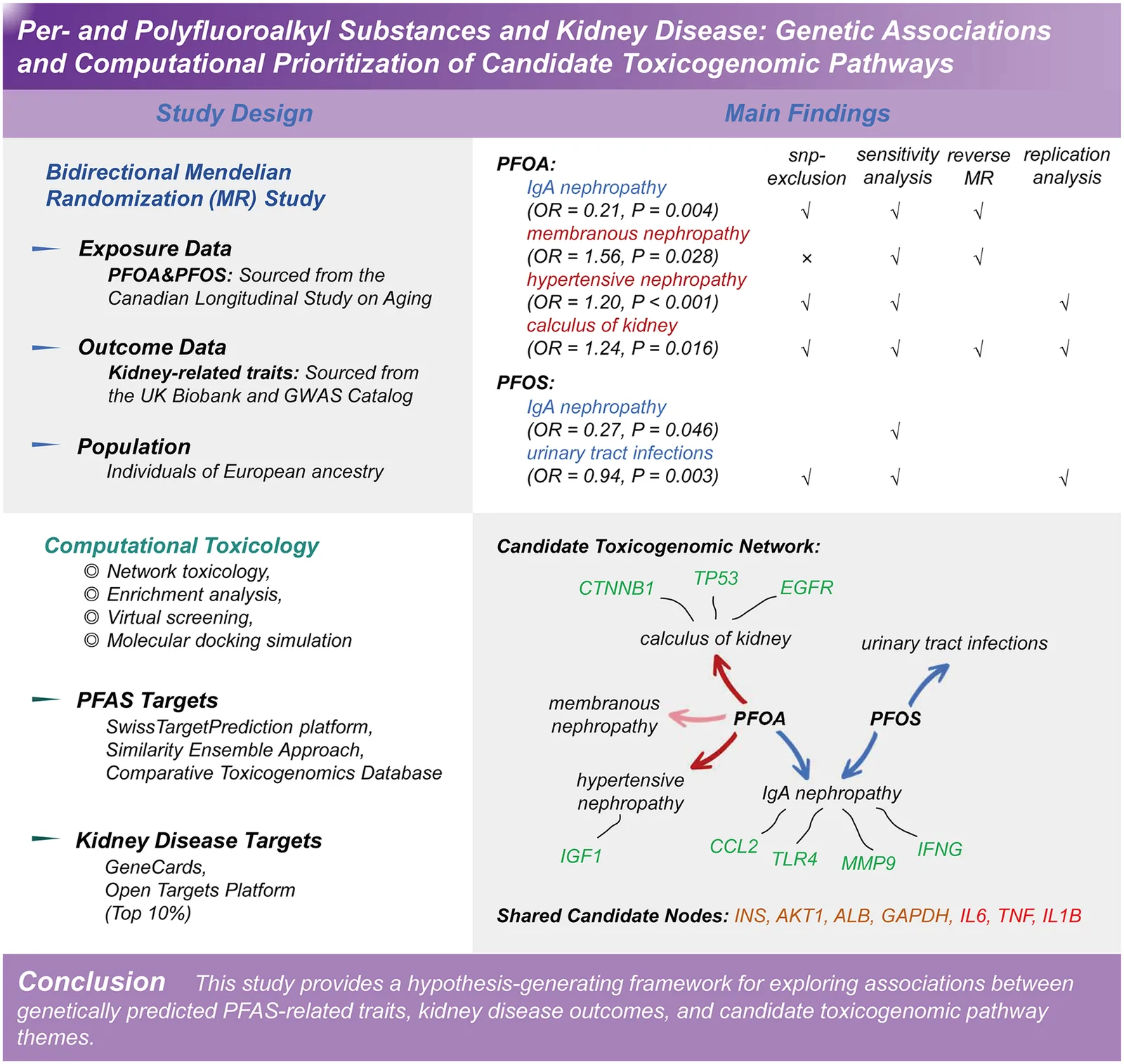
Flow chart of this study.

This study aims to investigate associations between genetically predicted circulating PFAS levels and kidney disease outcomes by integrating genetic epidemiology and computational toxicology approaches. Specifically, we explored the genetic associations of circulating PFOA and PFOS levels with five common kidney diseases. In parallel, computational toxicology analyses were performed to prioritize database-derived candidate molecular targets and biological pathway themes that may help generate testable hypotheses regarding PFAS–kidney disease associations. Molecular docking was further used to estimate the potential binding compatibility between PFOA/PFOS and selected network-prioritized candidate proteins. Through these analyses, we aimed to provide a hypothesis-generating framework for prioritizing candidate toxicogenomic pathways for future experimental validation (Fig 1).

## 2. Method

### 2.1. Data source for genetic epidemiology

Genetic instruments for genetically predicted circulating PFAS levels, specifically PFOA and PFOS, were obtained from a GWAS on plasma metabolites, which analyzed 1091 metabolites and 309 metabolite ratios in a cohort of 8299 Europeans from the Canadian Longitudinal Study on Aging (CLSA) [16]. The study identified genetic variants associated with PFAS levels in circulation through high-resolution metabolomics combined with genome-wide genotyping. Kidney disease outcomes, including IgA nephropathy, membranous nephropathy, hypertensive nephropathy, calculus of kidney, and urinary tract infections, were sourced from the UK Biobank and other large GWAS cohorts. All participants were of European descent, and genetic data were aligned with the HG19/GRCh37 genome build. Sample sizes for each trait differed across datasets, with details provided in Table 1.

**Table 1:** Details for the GWAS datasets.

GWAS ID	Trait	Author	Population	Year	Sample Size
GCST90200171	PFOA levels	Chen Y	European	2023	8199
GCST90200100	PFOS levels	Chen Y	European	2023	8218
ieu-a-1081	IgA nephropathy	Feehally J	European	2010	5957
ebi-a-GCST010005	Membranous nephropathy	Xie J	European	2020	7979
ukb-b-19955	Hypertensive nephropathy	Ben Elsworth	European	2018	463010
ukb-a-574	Calculus of kidney	Neale	European	2017	337199
ebi-a-GCST90013940	Urinary tract infection (SPA)	Mbatchou J	European	2021	397867
ebi-a-GCST90013890	Urinary tract infection (Firth)	Mbatchou J	European	2021	397867

### 2.2. Genetic instrumental variable selection

Genetic instruments for PFOA/PFOS exposure were chosen based on SNPs associated with their circulating levels, selected according to statistical significance thresholds and quality control procedures to obtain reliable and independent genetic proxies for circulating PFAS-related traits. Initial filtering involved applying a suggestive genome-wide significance threshold (*P* < 1 × 10^−5^), considering the relatively smaller sample size in the metabolomics GWAS [17]. To avoid confounding effects from linkage disequilibrium (LD), SNPs in the major histocompatibility complex (MHC) region (chromosome 6: 26–34 Mb) were excluded due to its complex LD structure [18]. The genetic instruments were further processed using PLINK version 1.90 (http://pngu.mgh.harvard.edu/purcell/plink/) with the 1000 Genomes Project European reference panel, ensuring independence through a clumping window of 10000 kb and an r² threshold of < 0.001 for LD. Moreover, SNPs with a minor allele frequency (MAF) ≤ 0.01 and palindromic SNPs were removed [19].

The strength of each instrumental variable was quantified using the F-statistic, calculated by


$$\begin{array}{c} F=\;R^{\text{2}}\;\times \frac{\left(N-\text{1}-K\right)}{\left(\text{1}-R^{\text{2}}\right)}\times K, \end{array}$$

where R² indicates the proportion of variance in PFAS levels explained by the genetic instrument, N is the sample size from the GWAS, and K represents the number of SNPs selected. SNPs with an F-statistic exceeding 10 were retained to mitigate potential bias from weak instruments [20]. The proportion of variance in PFAS levels explained by the SNPs was determined using the coefficient of determination R², calculated as


$$\begin{array}{c} R^{\text{2}}=\text{2}\;\times EAF\times (\text{1}-EAF)\times \beta^{\text{2}}, \end{array}$$

where EAF refers to the effect allele frequency, and β represents the genetic effect size on PFAS levels. Detailed SNP-level information, including effect alleles, effect sizes, standard errors, P values, R² values, and F-statistics, has been provided in S1 Table.

### 2.3. Bidirectional mendelian randomization and sensitivity analysis

Mendelian randomization (MR) was employed to explore the potential association between genetically predicted circulating PFAS levels and kidney diseases, utilizing genetic variants as instrumental variables to reduce biases from confounding and reverse causality.

To evaluate the MR assumptions and support interpretation of the MR estimates, the following were assessed: (1) relevance, confirming that the instrumental variables were strongly associated with the exposure; (2) independence, ensuring that the genetic instruments were not correlated with potential confounders; and (3) exclusion restriction, where the effects on the outcome were mediated solely through the exposure [11]. The RadialMR package was used to identify and exclude pleiotropic variants, while the PhenoScanner GWAS database (http://phenoscanner.medschl.cam.ac.uk) was queried to detect associations with potential confounders, including smoking, alcohol consumption, socioeconomic status, and blood pressure [21]. Proxies were selected with a linkage disequilibrium threshold of r² > 0.8, and SNPs associated with confounders were excluded [22]. MR Steiger filtering was also applied to evaluate the directionality of the association [23].

Sensitivity analyses were performed to assess pleiotropy and heterogeneity. MR-Egger regression was utilized to detect directional pleiotropy, with an intercept closer to zero indicating reduced pleiotropic bias [24]. Outliers were identified and corrected using MR-PRESSO, which evaluates the impact of horizontal pleiotropy on MR estimates [25]. Cochran’s Q test was used to evaluate heterogeneity among the genetic instruments [26], and a leave-one-out analysis was performed to check for the influence of any single SNP on the results.

The SNP-specific MR estimate was calculated using the Wald ratio, and the overall MR estimate was derived using inverse variance weighting (IVW), which provides a consistent estimate under standard instrumental-variable assumptions and in the absence of horizontal pleiotropy [27]. To ensure robustness against invalid instruments, the weighted median estimator was used, and MR-Egger regression was employed to adjust for directional pleiotropy under the Instrument Strength Independent of Direct Effect (InSIDE) assumption [28]. Maximum likelihood estimation (MLE) and MR robust adjusted profile score (MR-RAPS) were also applied as complementary MR methods [29,30]. Results were presented as odds ratios (OR) with 95% confidence intervals (CI), with statistical significance defined as *P* < 0.05.

To confirm the robustness of the MR estimates, the criteria outlined by Yoshiji et al. were applied: (1) no significant pleiotropy (MR-Egger & MR-PRESSO *P* > 0.05); (2) I² statistic < 50%, indicating low heterogeneity; (3) consistent results in the leave-one-out sensitivity analysis after outlier removal; and (4) non-significant reverse MR analysis (*P* > 0.05). This MR framework was also used for bidirectional analyses to evaluate potential reverse causation. All MR analyses were conducted in R version 4.3.2 using the TwoSampleMR package version 0.5.6, MendelianRandomization package version 0.9.0, RadialMR package version 1.1, MR-PRESSO package version 1.0, and mr.raps package version 0.4.2.

### 2.4. Data source for computational toxicology

For PFAS, we retrieved the chemical structures and SMILES formats of “perfluorooctanoic acid” and “perfluorooctane sulfonate” from PubChem (https://pubchem.ncbi.nlm.nih.gov/). Potential molecular targets were then predicted using the SwissTargetPrediction platform (http://www.swisstargetprediction.ch/) and the Similarity Ensemble Approach (SEA, https://sea.bkslab.org/). In addition, candidate targets with an interaction score > 1 were obtained from the Comparative Toxicogenomics Database (CTD, https://ctdbase.org/). Data from these three resources were integrated, duplicates were removed, and candidate PFAS-related target sets were compiled (S2 Table). For kidney diseases, candidate disease-associated targets were retrieved from the GeneCards (https://www.genecards.org/) and the Open Targets Platform (https://platform.opentargets.org/). The top 10% of targets were selected based on relevance scores and prioritization criteria, and the candidate disease-associated target sets were assembled following the same procedure (S3-S7 Tables). These disease-associated targets were derived from database annotations and literature-curated or predicted associations and were therefore considered disease-associated candidate targets.

### 2.5. PFAS–kidney disease candidate overlapping target screening and enrichment analysis

To identify candidate overlapping targets between PFAS-related candidate target sets and kidney disease-associated candidate target sets, PFAS-related candidate targets obtained from the above analyses were intersected with disease-associated candidate targets, and the overlapping targets were defined as PFAS–kidney disease candidate overlapping targets. To characterize the biological processes and pathway themes enriched among these candidate overlapping targets, Gene Ontology (GO) and Kyoto Encyclopedia of Genes and Genomes (KEGG) pathway enrichment analyses were performed using the clusterProfiler package version 4.8.3 [31]. GO analysis included three categories: biological process (BP), cellular component (CC), and molecular function (MF). KEGG analysis was conducted to explore enriched pathway themes. Visualization of the enrichment results was generated according to the statistical parameters, and pathways with an adjusted *P* < 0.05 were considered significantly enriched.

### 2.6. Construction of PPI networks and identification of network-topological hub targets

The candidate overlapping targets were submitted to the STRING database (http://www.string-db.org), restricting the species to Homo sapiens and setting the minimum required interaction score to medium confidence (0.400). These parameters were used to retain protein–protein interactions with at least medium confidence. The interaction results generated by STRING were then imported into Cytoscape software (version 3.8.2) for visualization and network analysis [32]. Network-topological hub targets were prioritized using the CytoHubba plugin, which ranks nodes according to network-topological properties through multiple topological algorithms, including Degree, Edge Percolated Component (EPC), Maximum Neighborhood Component (MNC), and Maximal Clique Centrality (MCC). The top 10 targets ranked by each algorithm were selected, and the overlapping nodes across all algorithms were defined as network-topological hub targets. To further support the network-level robustness of these hub targets, the Molecular Complex Detection (MCODE) algorithm was applied to identify densely connected modules within the PPI network, thereby enhancing the topological confidence of the final results.

### 2.7. MGI knockout/null mouse phenotype annotation of candidate targets

To provide additional functional phenotype context for the prioritized candidate targets, Mouse Genome Informatics (MGI) phenotypic allele and allele–phenotype reports were queried. Candidate genes were matched to mouse marker symbols, and targeted or endonuclease-mediated null/knockout alleles were prioritized. Reported phenotypes were screened for renal/urinary and immune/infection/inflammatory terms. This analysis was used as functional phenotype annotation to support candidate-target prioritization.

### 2.8. Molecular docking simulation

To estimate the in silico binding compatibility between PFAS compounds and selected network-prioritized candidate proteins, molecular docking analyses were conducted. The three-dimensional structures of selected candidate proteins in PDB format were retrieved from the RCSB Protein Data Bank (https://www.rcsb.org/), while the molecular structure files of PFOA and PFOS were obtained from PubChem (https://pubchem.ncbi.nlm.nih.gov/). Docking simulations were performed using CB-Dock2 (https://cadd.labshare.cn/cb-dock2/php/blinddock.php), and the results were further analyzed and visualized with R software (version 4.3.2) through heatmap representation. Binding affinity was evaluated based on binding energy values: more negative binding energy values were interpreted as indicating more favorable predicted binding in the docking model, with values <−5 kcal/mol considered suggestive of relatively favorable in silico binding.

To further evaluate the predicted relative binding preference of PFAS, additional control docking analyses were performed for the selected candidate proteins. Glycine and butyric acid were selected as representative non-PFAS control ligands, whereas octanoic acid was included as a non-fluorinated C8 carboxylic acid comparator for PFOA. These control ligands were docked to the same candidate protein structures using the same CB-Dock2 workflow as PFOA and PFOS.

## 3. Results

### 3.1. Genetic instrument selection and annotation for PFAS

After LD clumping, harmonization, and quality control, 57 independent SNPs were retained as instruments for genetically predicted circulating PFOA levels and 66 independent SNPs were retained for genetically predicted circulating PFOS levels. The cumulative variance explained by the retained instruments was 16.12% for PFOA and 18.56% for PFOS. The F-statistics ranged from 20.46 to 54.33 for PFOA and from 21.98 to 47.10 for PFOS, with mean F-statistics of 23.25 and 24.85, respectively. All retained SNPs had F-statistics greater than 10, suggesting that substantial weak instrument bias was unlikely. Detailed information on the selected instrumental SNPs, including effect alleles, effect sizes, standard errors, P values, R² values, and F-statistics, is provided in S1 Table.

Among these instruments, several lead variants showed relatively strong associations with circulating PFAS levels. For PFOA, rs35008345 showed the strongest association and was annotated to SLC22A11, which encodes organic anion transporter 4 (OAT4), a renal organic anion transporter involved in tubular handling of endogenous metabolites and xenobiotic organic anions [33–35]. For PFOS, the strongest instruments included rs141471965 and rs76713685; rs141471965 was located in an ABCG2-related locus. ABCG2 encodes an ATP-binding cassette efflux transporter involved in the disposition of urate, xenobiotics, and endogenous metabolites [36–38]. These lead variants suggest that genetically predicted circulating PFAS levels may partly reflect inherited differences in transporter-mediated distribution, renal handling, or elimination. However, most individual SNPs have not been functionally validated for PFAS toxicokinetics and should therefore be interpreted primarily as genetic proxies for circulating PFAS levels rather than as fully characterized mechanistic variants.

To further characterize the genetic instruments, we additionally annotated all independent PFAS-associated SNPs used in the MR analyses to their nearest genes or available eQTL target genes using Ensembl and GTEx resources. This annotation mapped the PFAS instruments to 55 unique protein-coding genes, indicating that the PFAS instruments were distributed across a broader set of loci rather than being restricted to SLC22A11 or ABCG2. Because some SNP-mapped genes may have reported kidney-related functions, we further summarized the basic molecular functions, kidney-related evidence context, variant-level OpenGWAS/PheWAS evidence, and interpretation regarding potential instrument-related pleiotropy of these mapped genes. Detailed SNP-level annotations and functional evidence stratification are provided in S1 Text and S8 and S9 Tables.

### 3.2. MR analysis of PFAS and kidney diseases

**Fig 2:**
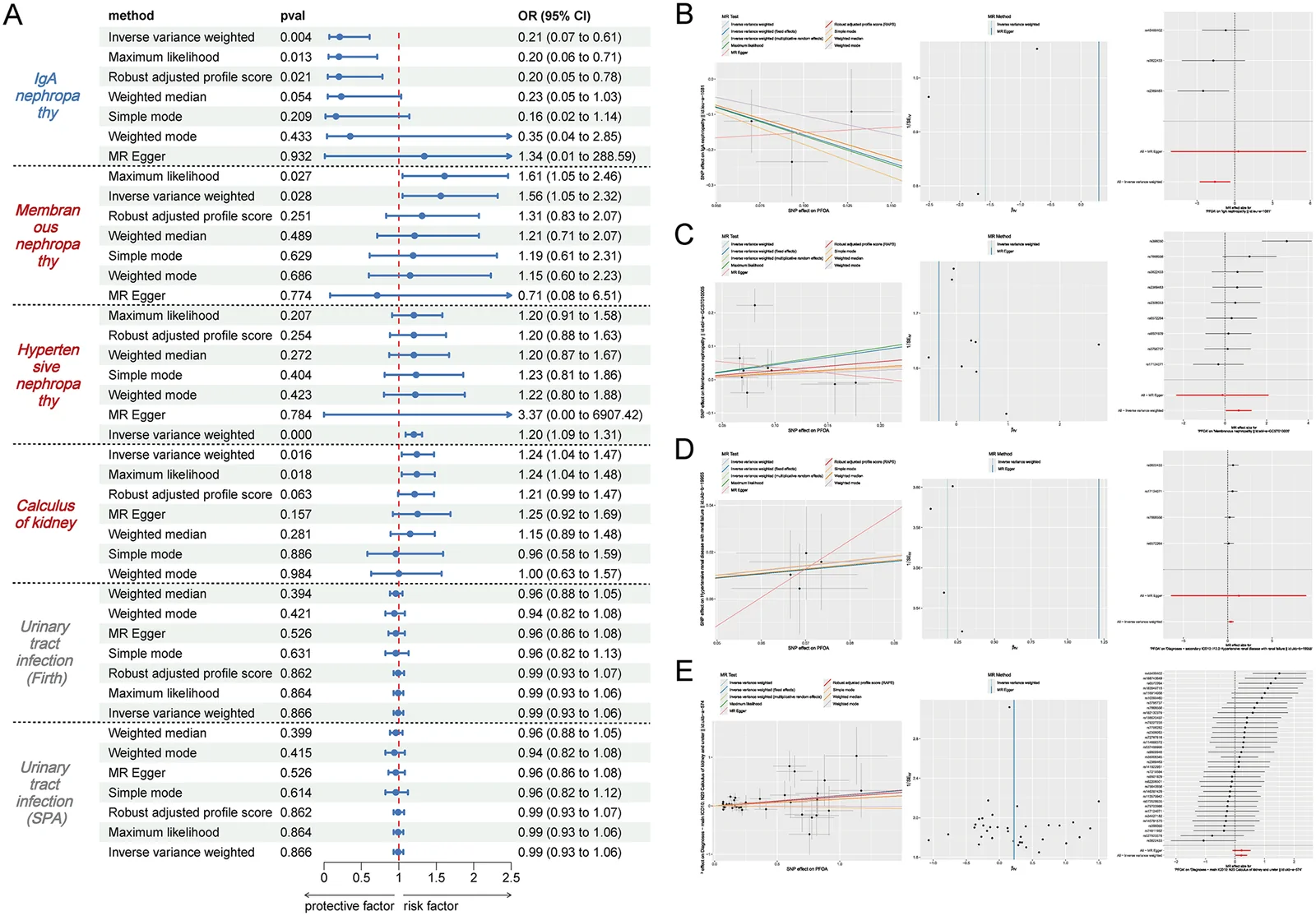
Detailed Mendelian randomization (MR) results for associations between PFOA and five kidney disease outcomes. **(A)** Forest plot for the significant associations between PFOA and five kidney disease outcomes, analyzed using multiple MR methods. **(B–E)** Tripartite plots for MR results: **(B)** IgA nephropathy, (C) membranous nephropathy, (D) hypertensive nephropathy, and (E) calculus of kidney. Each tripartite plot includes: (i) a scatter plot of MR results, (ii) a funnel plot for assessing potential bias, and (iii) a single SNP forest plot showing the SNP-specific MR estimate on the kidney disease outcome. The PFOA–membranous nephropathy association shown here represents the primary MR signal and was not retained after targeted SNP-exclusion analyses.

**Fig 3:**
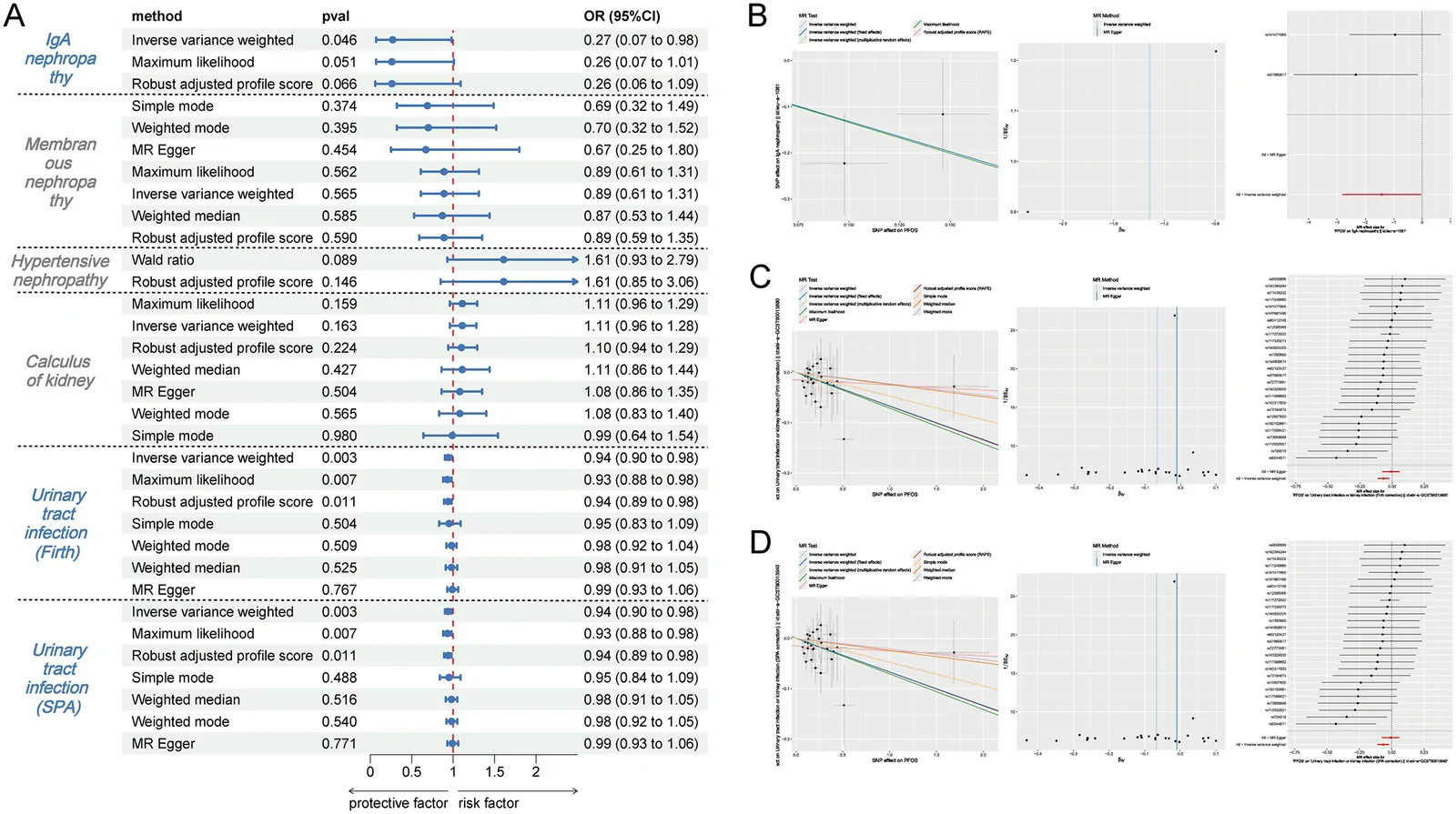
Detailed Mendelian randomization (MR) results for associations between PFOS and five kidney disease outcomes. **(A)** Forest plot for the significant associations between PFOS and five kidney disease outcomes, analyzed using multiple MR methods. **(B–D)** Tripartite plots for MR results: **(B)** IgA nephropathy, and (C, D) urinary tract infections. Each tripartite plot includes: (i) a scatter plot of MR results, (ii) a funnel plot for assessing potential bias, and (iii) a single SNP forest plot showing the SNP-specific MR estimate on the kidney disease outcome.

To estimate the associations between genetically predicted circulating PFOA/PFOS levels and kidney diseases, we performed MR analyses primarily using the inverse-variance weighted (IVW) method, supplemented by robust adjusted profile score (RAPS), maximum likelihood, MR-Egger, weighted median, weighted mode, and simple mode methods. Kidney disease outcomes included IgA nephropathy, membranous nephropathy, hypertensive nephropathy, calculus of kidney, and urinary tract infection. A comprehensive summary of the MR results is presented in Fig 2 for PFOA and Fig 3 for PFOS, summarizing the associations between genetically predicted circulating PFAS levels and five kidney disease outcomes.

In the primary MR analysis, genetically predicted elevated PFOA levels were inversely associated with IgA nephropathy (OR = 0.21, P = 0.004), but positively associated with membranous nephropathy (OR = 1.56, P = 0.028), hypertensive nephropathy (OR = 1.20, P < 0.001), and calculus of kidney (OR = 1.24, P = 0.016). Genetically predicted higher PFOS levels were inversely associated with IgA nephropathy (OR = 0.27, P = 0.046) and urinary tract infection (OR = 0.94, P = 0.003). These results were consistent across multiple MR methods. The scatter plots visualize the relationship between the genetic instruments and the outcomes; the funnel plots did not suggest obvious asymmetry; and the individual SNP forest plots display SNP-specific MR estimates and their contribution to the overall MR estimate. The robustness of these primary associations was further evaluated using sensitivity analyses and targeted SNP-exclusion analyses, as described below.

### 3.3. Sensitivity analysis and reverse MR analysis for PFAS and kidney diseases

To assess the robustness of our findings, we conducted sensitivity analyses. Heterogeneity was assessed using Cochran’s Q test for both IVW and MR-Egger methods, which showed no significant heterogeneity (*P* > 0.05 for all associations) (Tables 2, 3). Additionally, the MR-Egger intercept test and the MR-PRESSO Global test revealed no evidence of pleiotropy (*P* > 0.05 for all associations), indicating that the observed MR estimates were unlikely to be substantially biased by directional pleiotropy (Tables 2, 3). Furthermore, leave-one-out analysis demonstrated that no single SNP disproportionately influenced the overall results, as the MR estimates remained robust when each SNP was iteratively removed. To rule out reverse causation, we performed reverse MR analyses using kidney diseases as exposures and PFAS as outcomes (Tables 2, 3), which showed no significant reverse MR associations (*P* > 0.05 for all associations). Replication analysis using MR-PRESSO supported three significant associations: PFOA-hypertensive nephropathy, PFOA-calculus of kidney, and PFOS- urinary tract infection (Table 4). These analyses supported the robustness of several MR findings.

**Table 2:** Sensitivity analysis of PFOA to kidney diseases and reverse MR analysis.

Outcome	Heterogeneity				Pleiotropy			Reverse Causality			
	MR Egger		IVW		Egger-intercept	*P*	MR-Presso Global *P*	Steiger filtering	Reverse MR (IVW)		
	Q	*P*	Q	*P*					Beta	SE	*P*
IgA nephropathy	1.09	0.296	1.63	0.442	-0.181	0.610	0.066	√	0.00	0.02	0.754
Membranous nephropathy	18.22	0.112	19.58	0.152	0.070	0.493	0.066	√			
Hypertensive nephropathy	0.25	0.884	0.32	0.957	-0.072	0.815	0.957	√	22.78	18.62	0.221
Calculus of kidney	43.04	0.113	43.05	0.137	-0.002	0.925	0.158	√	0.55	2.41	0.820
Urinary tract infection (Firth)	7.80	0.955	8.24	0.961	0.005	0.515	0.958	√			
Urinary tract infection (SPA)	7.80	0.955	8.24	0.961	0.005	0.515	0.969	√			

**Table 3:** Sensitivity analysis of PFOS to kidney diseases and reverse MR analysis.

Outcome	Heterogeneity				Pleiotropy			Reverse Causality			
	MR Egger		IVW		Egger-intercept	*P*	MR-Presso Global *P*	Steiger filtering	Reverse MR (IVW)		
	Q	*P*	Q	*P*					Beta	SE	*P*
IgA nephropathy			1.15	0.283			0.336	√	0.00	0.02	0.754
Membranous nephropathy	3.61	0.823	4.00	0.857	0.037	0.555	0.871	√			
Hypertensive nephropathy								√	-1.61	17.62	0.927
Calculus of kidney	43.57	0.283	43.68	0.318	0.008	0.756	0.314	√	5.00	4.64	0.281
Urinary tract infection (Firth)	19.36	0.779	24.30	0.559	-0.015	0.136	0.518	√			
Urinary tract infection (SPA)	19.35	0.780	24.30	0.559	-0.015	0.135	0.506	√			

**Table 4:** Replication analysis using MR-PRESSO.

Exposure	Outcome	MR Analysis	Effect estimate	*P*	OR	OR_down	OR_up
PFOA	Hypertensive nephropathy	Raw	0.18	0.030	1.20	1.09	1.31
PFOA	Calculus of kidney	Raw	0.21	0.040	1.24	1.02	1.50
PFOS	Urinary tract infection (Firth)	Raw	-0.07	0.006	0.94	0.90	0.98
PFOS	Urinary tract infection (SPA)	Raw	-0.07	0.006	0.94	0.90	0.98

To further address potential instrument-related pleiotropy arising from renal transport or kidney-related traits, we performed additional targeted SNP-exclusion sensitivity analyses. These analyses were conducted after excluding SLC22A11/ABCG2-related lead SNPs, after excluding SNPs associated with kidney-related or kidney-adjacent traits in OpenGWAS/PheWAS, and after excluding both SNP sets. After these targeted exclusions, the PFOA–IgA nephropathy, PFOA–hypertensive nephropathy, and PFOS–urinary tract infection associations remained statistically significant, whereas the PFOA–calculus of kidney association remained directionally consistent but was attenuated after excluding kidney-related PheWAS SNPs. The PFOS–IgA nephropathy association was not estimable under the targeted exclusion framework because no valid instrument remained after exclusion. Importantly, the PFOA–membranous nephropathy association was not retained after targeted SNP-exclusion analyses. After excluding the SLC22A11/ABCG2-related lead SNPs, the association was no longer statistically significant (β = 0.444, P = 0.160). Similar attenuation was observed after excluding kidney-related OpenGWAS/PheWAS SNPs or both SNP sets (β = 0.157, P = 0.292). These results indicate that the primary PFOA–membranous nephropathy association was sensitive to instrument selection and should not be interpreted as an established causal association in this study. In addition, some statistically significant associations had small estimated effect sizes, particularly PFOA–hypertensive nephropathy and PFOA–calculus of kidney. Therefore, these findings should be interpreted as modest genetic associations rather than evidence of large individual-level clinical effects. Detailed SNP annotation, OpenGWAS/PheWAS screening results, exclusion status, and MR estimates are provided in S1 Text and S8–S14 Tables.

### 3.4 Candidate target screening and enrichment analysis of PFOA-IgA nephropathy network

**Fig 4:**
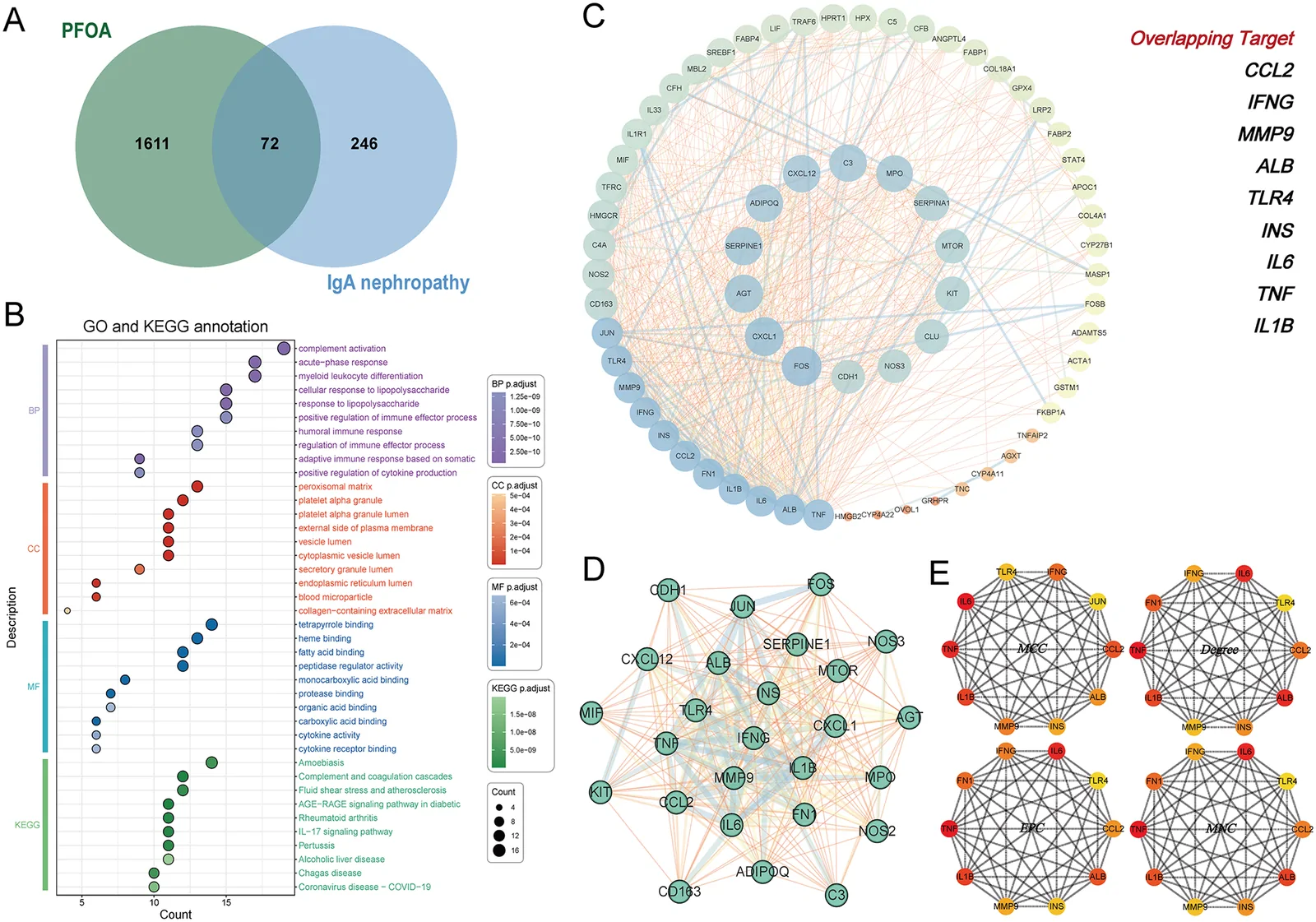
Candidate target and enrichment map of the PFOA-IgA nephropathy network. **(A)** Venn diagram analysis identified 72 candidate overlapping targets between PFOA-related and IgA nephropathy-associated target sets. **(B)** Gene Ontology (GO) and Kyoto Encyclopedia of Genes and Genomes (KEGG) pathway enrichment analysis. **(C)** Protein-protein interaction (PPI) network. **(D)** Densely connected modules within the PPI network using the Molecular Complex Detection (MCODE) algorithm. **(E)** Top ten network-ranked targets identified using the Degree, Edge Percolated Component (EPC), Maximum Neighborhood Component (MNC), and Maximal Clique Centrality (MCC) algorithms.

We retrieved the standard structure of PFOA from the PubChem database. After merging and removing duplicates, 1683 PFOA-related candidate targets were retrieved or predicted from the SwissTargetPrediction, SEA, and CTD databases. Additionally, 318 IgA nephropathy-associated candidate targets were retrieved from GeneCards and the Open Targets Platform. A Venn diagram analysis identified 72 candidate overlapping targets between PFOA-related and IgA nephropathy-associated target sets (Fig 4A). These 72 candidate overlapping targets were subsequently subjected to enrichment analysis.

Gene Ontology (GO) analysis showed that the top enriched biological process (BP) terms included immune-related processes such as “complement activation” and “acute-phase response.” In the cellular component (CC) category, the top terms included “platelet alpha granule” and “collagen-containing extracellular matrix.” Molecular function (MF) enrichment highlighted “fatty acid binding” and “cytokine receptor binding.” Kyoto Encyclopedia of Genes and Genomes (KEGG) pathway analysis showed that the candidate overlapping targets were enriched in pathway annotations including “complement and coagulation cascades,” “AGE-RAGE signaling in diabetes,” and “IL-17 signaling.” Together, these enrichment results indicate that the PFOA–IgA nephropathy candidate network was mainly characterized by complement/coagulation-related immune activation, cytokine-receptor- and IL-17-associated inflammatory signaling, and extracellular-matrix-associated response patterns (Fig 4B).

A protein-protein interaction (PPI) network was constructed for these candidate overlapping targets based on the STRING database, and the network topology was analyzed and visualized using Cytoscape version 3.8.2 (Fig 4C). To characterize densely connected modules within the PPI network, the Molecular Complex Detection (MCODE) algorithm was employed (Fig 4D). The top ten network-ranked targets were selected using several topological algorithms, including Degree, Edge Percolated Component (EPC), Maximum Neighborhood Component (MNC), and Maximal Clique Centrality (MCC) (Fig 4E). The overlapping targets identified across all algorithms were defined as network-topological hub targets, including CCL2, IFNG, MMP9, ALB, TLR4, INS, IL6, TNF, and IL1B.

### 3.5. Exploratory candidate target analysis of the primary PFOA–membranous nephropathy signal

Because the primary PFOA–membranous nephropathy association was not retained after targeted SNP-exclusion analyses, the following candidate target and enrichment analysis should be interpreted only as an exploratory computational summary of the primary MR signal. This analysis was retained for transparency but should not be considered evidence of a robust association, an established causal relationship, or a mechanistic pathway linking PFOA to membranous nephropathy.

**Fig 5:**
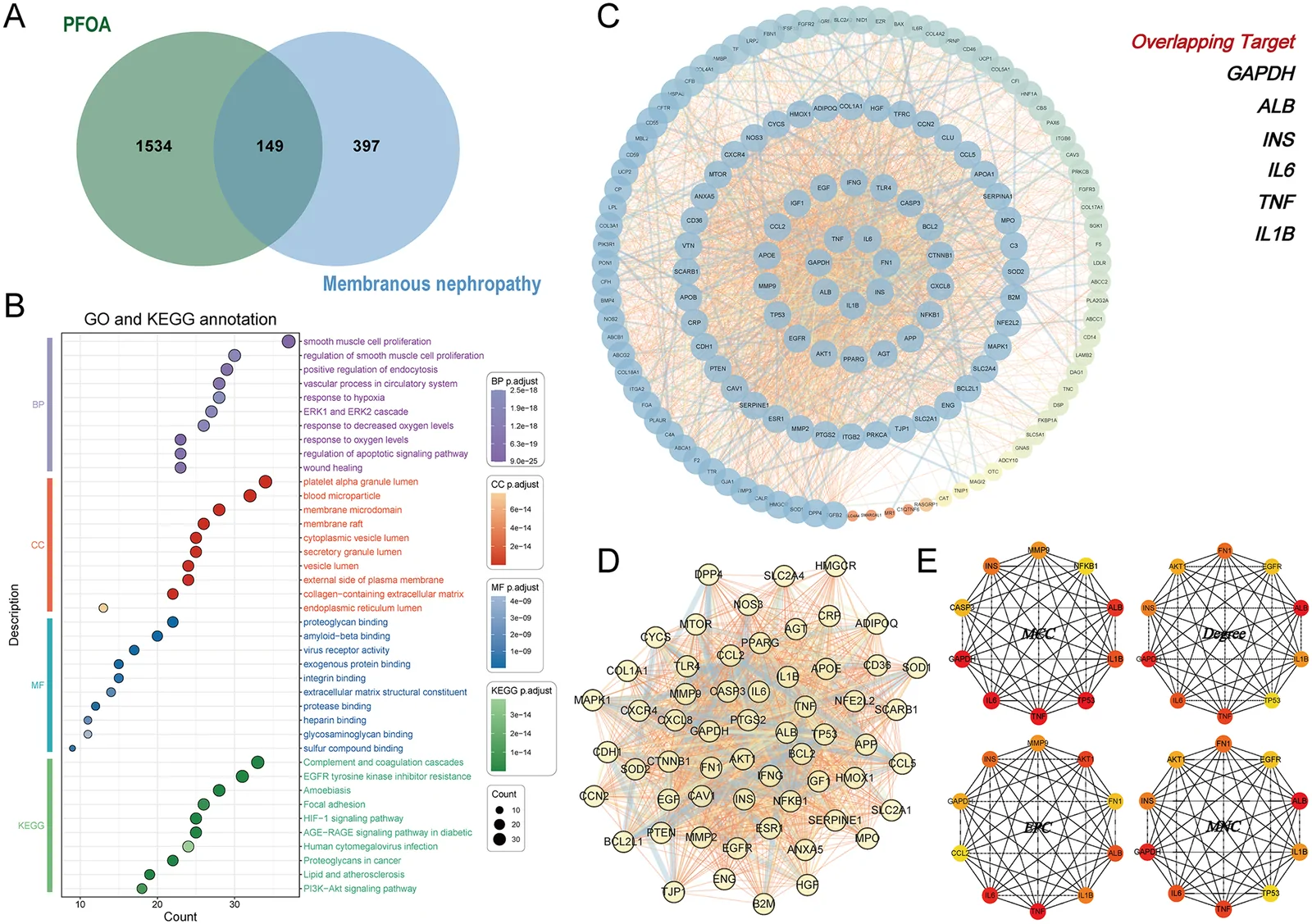
Exploratory candidate target and enrichment map of the primary PFOA–membranous nephropathy signal. **(A)** Venn diagram analysis identified 149 candidate overlapping targets between PFOA-related and membranous nephropathy-associated target sets. **(B)** GO and KEGG pathway enrichment analysis. **(C)** Protein-protein interaction (PPI) network. **(D)** Densely connected modules within the PPI network using the MCODE algorithm. **(E)** Top ten network-ranked targets identified using the Degree, EPC, MNC, and MCC algorithms.

After merging and removing duplicates, we retrieved 546 membranous nephropathy-associated candidate targets from GeneCards and the Open Targets platform. After merging with 1683 PFOA-related candidate targets from the SwissTargetPrediction, SEA, and CTD databases, Venn diagram analysis identified 149 candidate overlapping targets between PFOA-related and membranous nephropathy-associated target sets (Fig 5A). These 149 candidate overlapping targets were subsequently subjected to enrichment analysis.

GO analysis showed that the top enriched BP terms included “response to hypoxia” and “response to decreased oxygen levels.” In the CC category, the enriched terms centered on “platelet alpha granule lumen” and “collagen-containing extracellular matrix.” MF enrichment highlighted “fatty acid binding” and “cytokine receptor binding.” KEGG pathway analysis showed that the candidate overlapping targets were enriched in pathway annotations including “complement and coagulation cascades,” “EGFR tyrosine kinase inhibitor resistance,” and “HIF-1 signaling pathway.” In this exploratory analysis, these enrichment results suggested that the PFOA–membranous nephropathy candidate network was mainly characterized by oxygen-level/hypoxia-response annotations, complement/coagulation- and cytokine-receptor-related immune signaling, and extracellular-matrix-associated components (Fig 5B).

A PPI network was constructed for these candidate overlapping targets based on the STRING database, and the network topology was analyzed and visualized using Cytoscape version 3.8.2 (Fig 5C). To characterize densely connected modules within the PFOA–membranous nephropathy PPI network, the MCODE algorithm was employed (Fig 5D). The top ten network-ranked targets were selected using several topological algorithms, including Degree, EPC, MNC, and MCC (Fig 5E). The overlapping targets identified across all algorithms were defined as network-topological hub targets, including GAPDH, ALB, INS, IL6, TNF, and IL1B. Therefore, this network should be interpreted as an exploratory summary of the primary MR signal rather than evidence supporting a robust PFOA–membranous nephropathy association, and it was not used to support the main conclusions or mechanistic inference of this study.

### 3.6. Candidate target screening and enrichment analysis of PFOA-hypertensive nephropathy network

**Fig 6:**
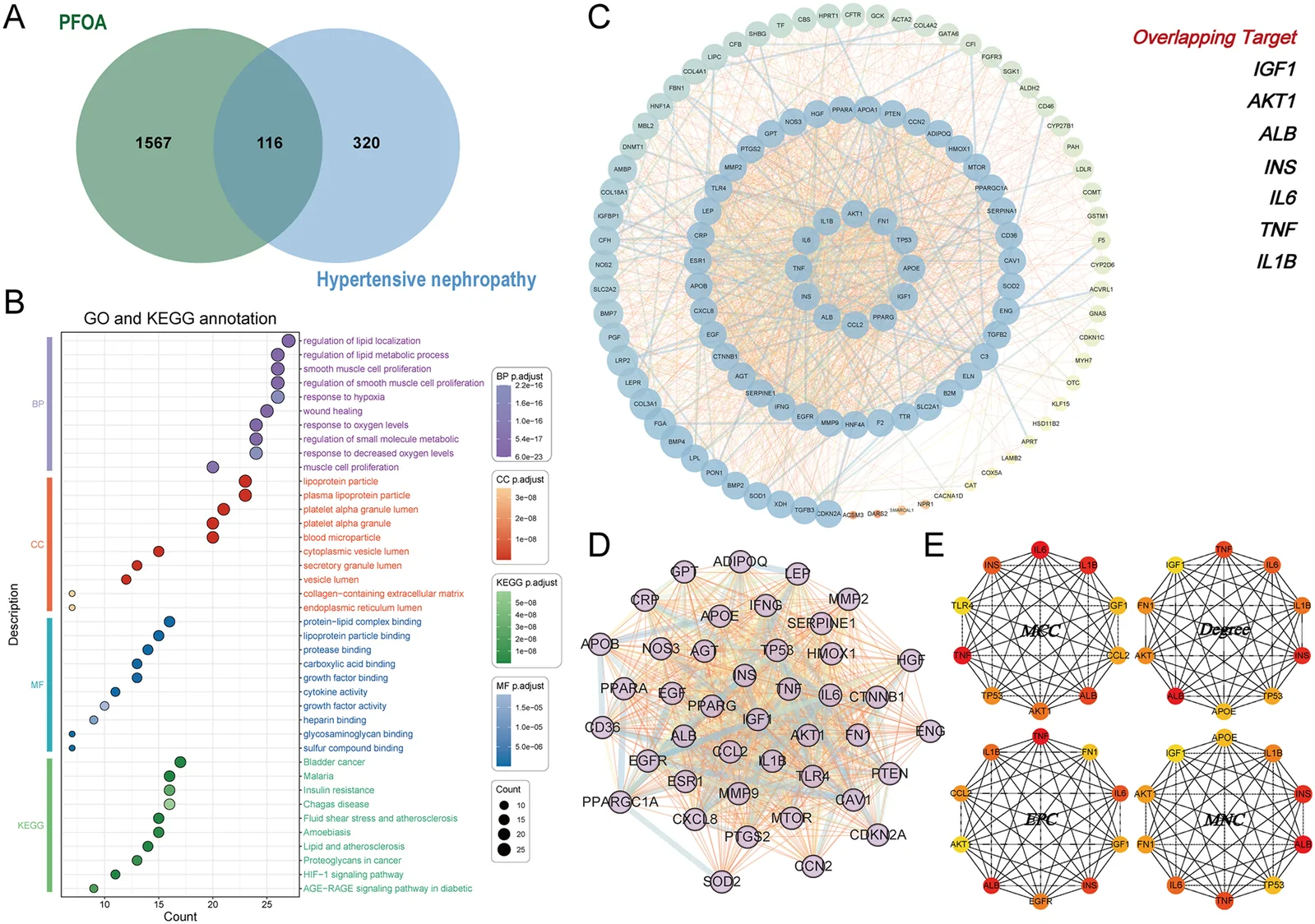
Candidate target and enrichment map of the PFOA-hypertensive nephropathy network. **(A)** Venn diagram analysis identified 116 candidate overlapping targets between PFOA-related and hypertensive nephropathy-associated target sets. **(B)** GO and KEGG pathway enrichment analysis. **(C)** Protein-protein interaction (PPI) network. **(D)** Densely connected modules within the PPI network using the MCODE algorithm. **(E)** Top ten network-ranked targets identified using the Degree, EPC, MNC, and MCC algorithms.

After merging and removing duplicates, we retrieved 436 hypertensive nephropathy-associated candidate targets from GeneCards and the Open Targets platform. After merging with 1683 PFOA-related candidate targets from the SwissTargetPrediction, SEA, and CTD databases, Venn diagram analysis identified 116 candidate overlapping targets between PFOA-related and hypertensive nephropathy-associated target sets (Fig 6A). These 116 candidate overlapping targets were subsequently subjected to enrichment analysis.

GO analysis showed that the top enriched BP terms included “smooth muscle cell proliferation,” “response to oxygen levels,” “response to hypoxia,” “response to decreased oxygen levels,” and “wound healing.” In the CC category, the enriched terms centered on “platelet alpha granules,” “plasma lipoprotein particles,” and the “extracellular matrix.” MF enrichment highlighted “protein-lipid complex binding,” “lipoprotein particle binding,” and “cytokine receptor activity.” KEGG pathway analysis showed that the candidate overlapping targets were enriched in pathway annotations including “malaria,” “insulin resistance,” “Chagas disease,” “atherosclerosis,” “HIF-1 signaling pathway,” “AGE-RAGE signaling in diabetes,” and “proteoglycans in cancer.” Together, these enrichment results indicate that the PFOA–hypertensive nephropathy candidate network was mainly characterized by vascular remodeling- and hypoxia-response-related processes, lipid/metabolic annotations, extracellular-matrix-associated components, and cytokine-receptor-related immune signaling (Fig 6B).

A PPI network was constructed for these candidate overlapping targets based on the STRING database, and the network topology was analyzed and visualized using Cytoscape version 3.8.2 (Fig 6C). To characterize densely connected modules within the PFOA–hypertensive nephropathy PPI network, the MCODE algorithm was employed (Fig 6D). The top ten network-ranked targets were selected using several topological algorithms, including Degree, EPC, MNC, and MCC (Fig 6E). The overlapping targets identified across all algorithms were defined as network-topological hub targets, including IGF1, AKT1, ALB, INS, IL6, TNF, and IL1B.

### 3.7. Candidate target screening and enrichment analysis of PFOA-calculus of kidney network

**Fig 7:**
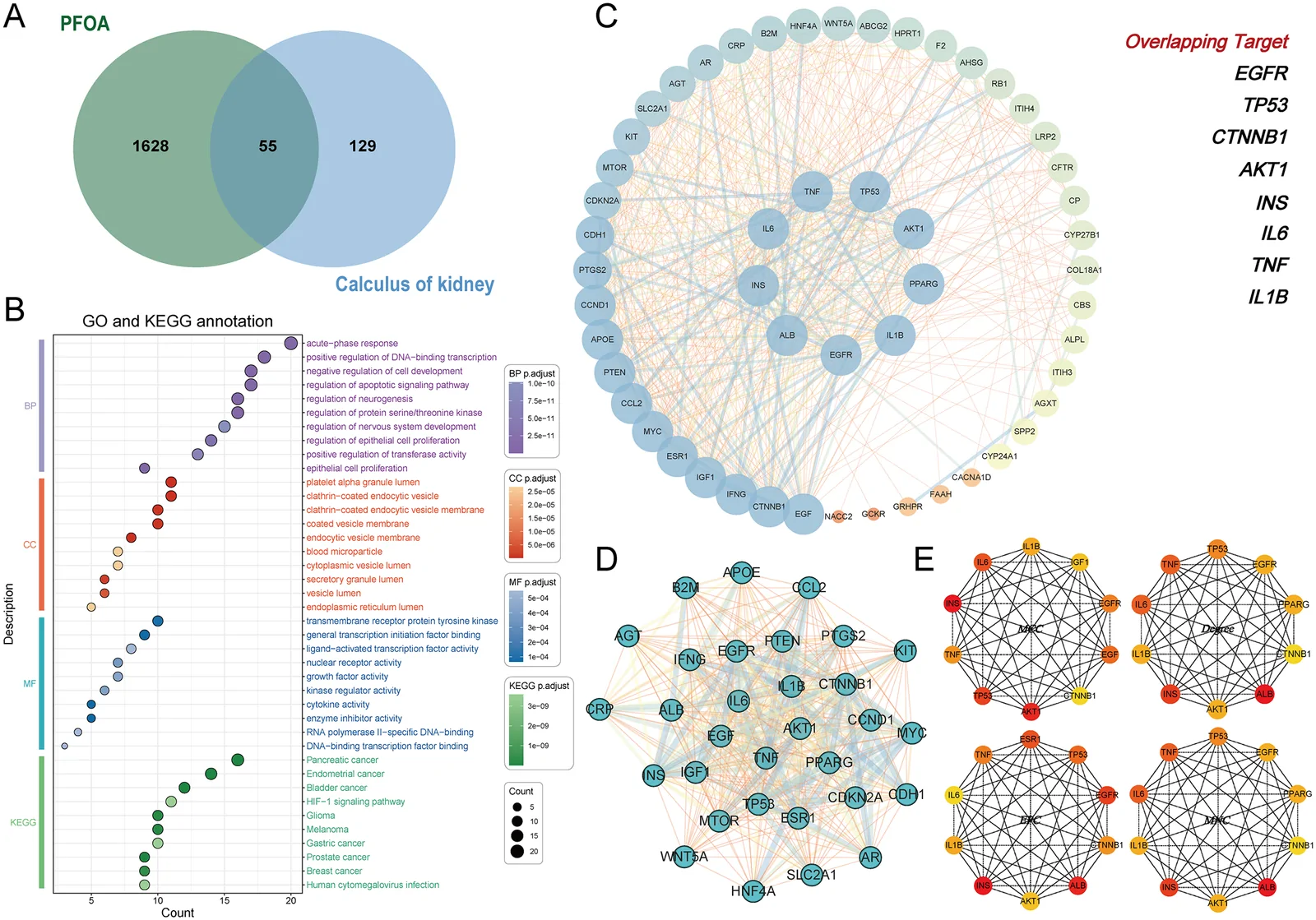
Candidate target and enrichment map of the PFOA-calculus of kidney network. **(A)** Venn diagram analysis identified 55 candidate overlapping targets between PFOA-related and calculus of kidney-associated target sets. **(B)** GO and KEGG pathway enrichment analysis. **(C)** Protein-protein interaction (PPI) network. **(D)** Densely connected modules within the PPI network using the MCODE algorithm. **(E)** Top ten network-ranked targets identified using the Degree, EPC, MNC, and MCC algorithms.

After merging and removing duplicates, we retrieved 184 calculus of kidney-associated candidate targets from GeneCards and the Open Targets platform. After merging with 1683 PFOA-related candidate targets from the SwissTargetPrediction, SEA, and CTD databases, Venn diagram analysis identified 55 candidate overlapping targets between PFOA-related and calculus of kidney-associated target sets (Fig 7A). These 55 candidate overlapping targets were subsequently subjected to enrichment analysis.

GO analysis showed that the top enriched BP terms included “acute-phase response,” “positive regulation of DNA-binding transcription,” and “regulation of apoptotic signaling pathway,” with additional terms related to cell development and neurogenesis. In the CC category, the enriched terms centered on “platelet alpha granules,” “clathrin-coated endocytic vesicles,” and “blood microparticles.” MF enrichment highlighted receptor- and transcription-factor-related terms, including “transmembrane receptor protein tyrosine kinase,” “ligand-activated transcription factor activity,” and “growth factor activity.” KEGG pathway analysis showed that the candidate overlapping targets were enriched in pathway annotations including “pancreatic cancer,” “endometrial cancer,” “bladder cancer,” “HIF-1 signaling pathway,” “glioma,” and “melanoma.” Together, these enrichment results indicate that the PFOA–calculus of kidney candidate network was mainly characterized by acute-phase and apoptotic-response terms, vesicle- and blood-microparticle-related cellular components, receptor/transcription-factor/growth-factor-related molecular functions, and HIF-1 signaling and cancer-related KEGG pathway annotations (Fig 7B).

A PPI network was constructed for these candidate overlapping targets based on the STRING database, and the network topology was analyzed and visualized using Cytoscape version 3.8.2 (Fig 7C). To characterize densely connected modules within the PFOA–calculus of kidney PPI network, the MCODE algorithm was employed (Fig 7D). The top ten network-ranked targets were selected using several topological algorithms, including Degree, EPC, MNC, and MCC (Fig 7E). The overlapping targets identified across all algorithms were defined as network-topological hub targets, including EGFR, TP53, CTNNB1, AKT1, INS, IL6, TNF, and IL1B.

### 3.8. Candidate target screening and enrichment analysis of PFOS-IgA nephropathy network

**Fig 8:**
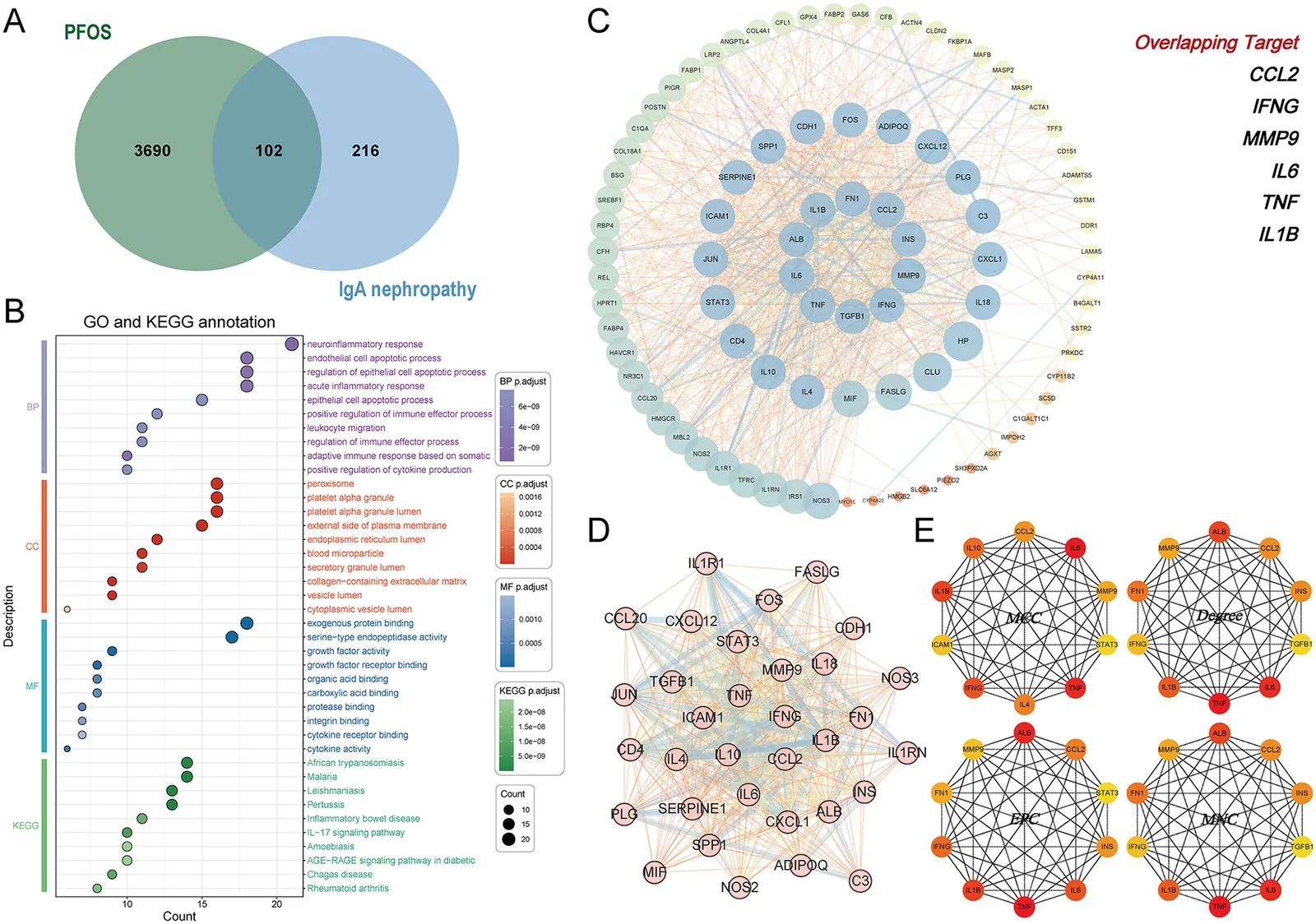
Candidate target and enrichment map of the PFOS-IgA nephropathy network. **(A)** Venn diagram analysis identified 102 candidate overlapping targets between PFOS-related and IgA nephropathy-associated target sets. **(B)** GO and KEGG pathway enrichment analysis. **(C)** Protein-protein interaction (PPI) network. **(D)** Densely connected modules within the PPI network using the MCODE algorithm. **(E)** Top ten network-ranked targets identified using the Degree, EPC, MNC, and MCC algorithm.

We retrieved the standard structure of PFOS from the PubChem database. After merging and removing duplicates, 3792 PFOS-related candidate targets were retrieved or predicted from the SwissTargetPrediction, SEA, and CTD databases. Additionally, 318 IgA nephropathy-associated candidate targets were retrieved from GeneCards and the Open Targets Platform. A Venn diagram analysis identified 102 candidate overlapping targets between PFOS-related and IgA nephropathy-associated target sets (Fig 8A). These 102 candidate overlapping targets were subsequently subjected to enrichment analysis.

GO analysis showed that the top enriched BP terms included “neuroinflammatory response,” “acute inflammatory response,” and “positive regulation of immune effector process,” with additional terms related to apoptosis and leukocyte migration. In the CC category, the enriched terms centered on “platelet alpha granule,” “peroxisome,” and “collagen-containing extracellular matrix.” MF enrichment highlighted “exogenous protein binding,” “serine-type endopeptidase activity,” and “cytokine receptor binding.” KEGG pathway analysis showed that the candidate overlapping targets were enriched in pathway annotations including “Malaria,” “Leishmaniasis,” “Pertussis,” “IL-17 signaling pathway,” “AGE-RAGE signaling in diabetes,” and “inflammatory bowel disease.” Together, these enrichment results indicate that the PFOS–IgA nephropathy candidate network was mainly characterized by inflammatory immune activation, immune-effector and leukocyte-response patterns, cytokine-receptor/IL-17-related inflammatory signaling, and extracellular-matrix-associated cellular contexts (Fig 8B).

A PPI network was constructed for these candidate overlapping targets based on the STRING database, and the network topology was analyzed and visualized using Cytoscape version 3.8.2 (Fig 8C). To characterize densely connected modules within the PFOS–IgA nephropathy PPI network, the MCODE algorithm was employed (Fig 8D). The top ten network-ranked targets were selected using several topological algorithms, including Degree, EPC, MNC, and MCC (Fig 8E). The overlapping targets identified across all algorithms were defined as network-topological hub targets, including CCL2, IFNG, MMP9, IL6, TNF, and IL1B.

### 3.9. Candidate target screening and enrichment analysis of PFOS-urinary tract infections network

**Fig 9:**
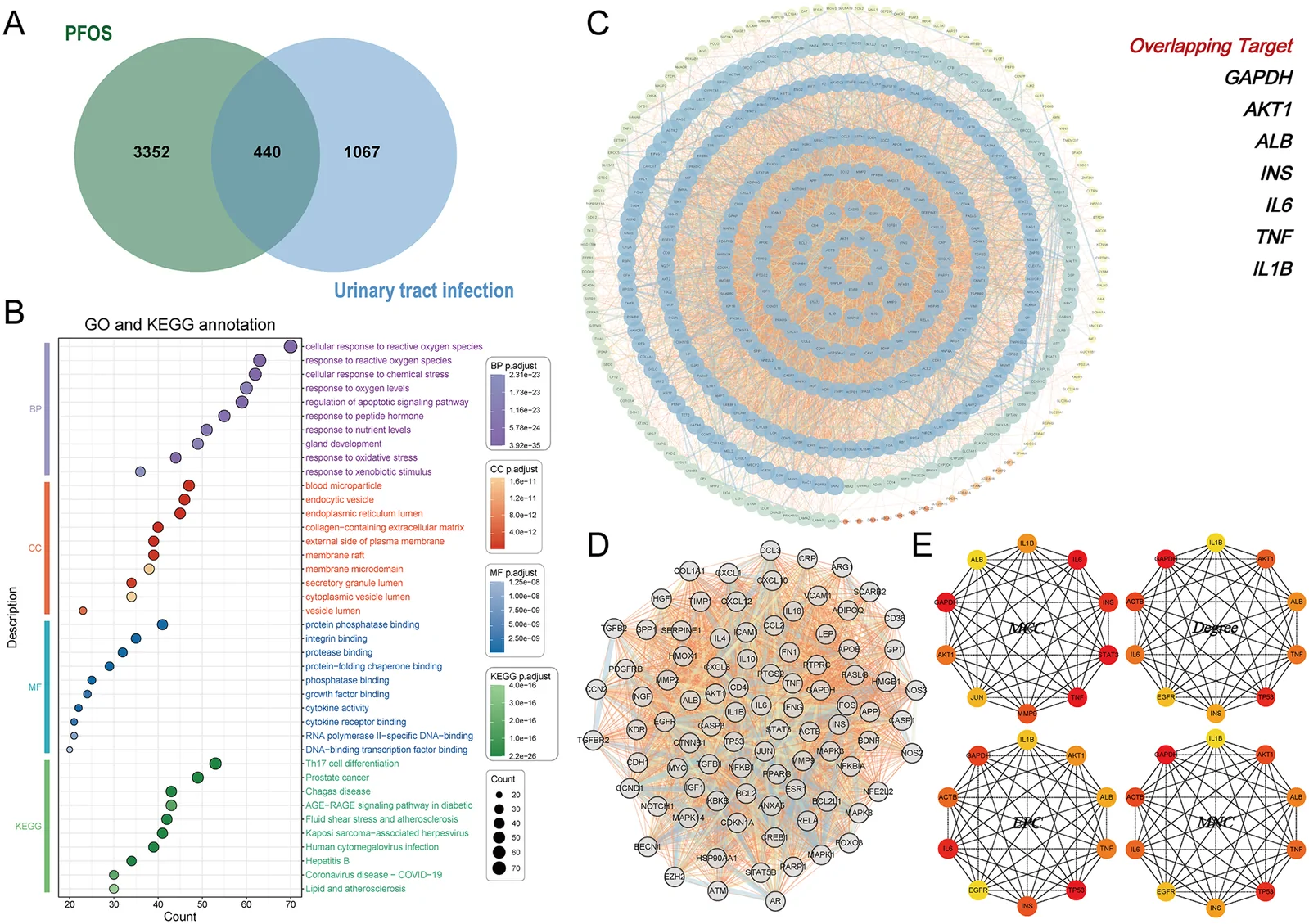
Candidate target and enrichment map of the PFOS-urinary tract infection network. **(A)** Venn diagram analysis identified 440 candidate overlapping targets between PFOS-related and urinary tract infection-associated target sets. **(B)** GO and KEGG pathway enrichment analysis. **(C)** Protein-protein interaction (PPI) network. **(D)** Densely connected modules within the PPI network using the MCODE algorithm. **(E)** Top ten network-ranked targets identified using the Degree, EPC, MNC, and MCC algorithm.

After merging and removing duplicates, we retrieved 1507 urinary tract infection-associated candidate targets from GeneCards and the Open Targets platform. After merging with 3792 PFOS-related candidate targets from the SwissTargetPrediction, SEA, and CTD databases, Venn diagram analysis identified 440 candidate overlapping targets between PFOS-related and urinary tract infection-associated target sets (Fig 9A). These 440 candidate overlapping targets were subsequently subjected to enrichment analysis.

GO analysis showed that the top enriched BP terms included “response to reactive oxygen species,” “response to oxidative stress,” and “regulation of apoptotic signaling pathway,” with additional terms related to cell proliferation and immune regulation. In the CC category, the enriched terms centered on “platelet alpha granule,” “collagen-containing extracellular matrix,” and “endoplasmic reticulum lumen.” MF enrichment highlighted “protein phosphatase binding,” “integrin binding,” and “cytokine receptor binding.” KEGG pathway analysis showed that the candidate overlapping targets were enriched in pathway annotations including “Prostate cancer,” “Chagas disease,” and “AGE-RAGE signaling pathway in diabetes.” Together, these enrichment results suggest that the PFOS–urinary tract infection candidate network was mainly characterized by cellular stress and apoptotic-response processes, extracellular-matrix and adhesion-related components, and cytokine-receptor-associated immune signaling (Fig 9B).

A PPI network was constructed for these candidate overlapping targets based on the STRING database, and the network topology was analyzed and visualized using Cytoscape version 3.8.2 (Fig 9C). To characterize densely connected modules within the PFOS–urinary tract infection PPI network, the MCODE algorithm was employed (Fig 9D). The top ten network-ranked targets were selected using several topological algorithms, including Degree, EPC, MNC, and MCC (Fig 9E). The overlapping targets identified across all algorithms were defined as network-topological hub targets, including GAPDH, AKT1, ALB, INS, IL6, TNF, and IL1B.

### 3.10. Literature and MGI phenotype context for network-topological hub targets

To further characterize the renal/urinary and functional phenotype context of the network-topological hub targets, we summarized literature-supported kidney-related evidence and performed knockout/null mouse phenotype annotation using Mouse Genome Informatics (MGI). The prioritized hub genes from the PFAS–kidney disease networks were mapped to mouse orthologous markers, and targeted or endonuclease-mediated knockout/null allele records were screened for renal/urinary and immune/infection/inflammatory phenotypes. In parallel, literature-supported evidence was summarized for each prioritized candidate target, focusing on renal/urinary disease, glomerular inflammation, tubular epithelial injury, kidney fibrosis, metabolic kidney disease, and immune-inflammatory contexts.

The integrated literature and MGI annotation showed that several network-topological hub targets had renal/urinary, kidney-injury, or immune-inflammatory contextual evidence (S15 Table). Among the prioritized candidates in the PFOA/PFOS–IgA nephropathy networks, CCL2, TLR4, MMP9, and IFNG were supported by IgA nephropathy, glomerulonephritis, mesangial inflammation, macrophage recruitment, or extracellular-matrix-remodeling evidence. In the PFOA–hypertensive nephropathy network, IGF1 was supported mainly by renal growth, renal hypertrophy, and diabetic kidney disease-context evidence. In the PFOA–calculus of kidney network, CTNNB1 and TP53 were supported by renal epithelial injury, oxalate/crystal-related injury, oxidative-stress, or ferroptosis-related evidence, whereas EGFR was supported mainly by broader renal injury, tubular repair, and fibrosis-related evidence. IL6, TNF, and IL1B appeared as shared inflammatory nodes across multiple networks and were supported mainly by renal inflammatory injury, immune-mediated nephropathy, and cytokine-response evidence. Broadly connected nodes such as INS, ALB, and GAPDH were interpreted as metabolic, biomarker-related, or housekeeping/network-topological context rather than disease-specific or PFAS-specific mechanistic targets.

These literature and MGI annotations provided additional renal/urinary and functional phenotype context for interpreting the network-prioritized candidate targets and for prioritizing proteins for subsequent molecular docking analysis. However, these annotations should not be interpreted as evidence of one-to-one target–disease causality or PFAS-mediated regulation. Instead, they support the biological relevance of selected candidate targets and provide a contextual basis for future experimental validation.

### 3.11. Integrated candidate network and molecular docking simulation of PFAS and kidney disease outcomes

**Fig 10:**
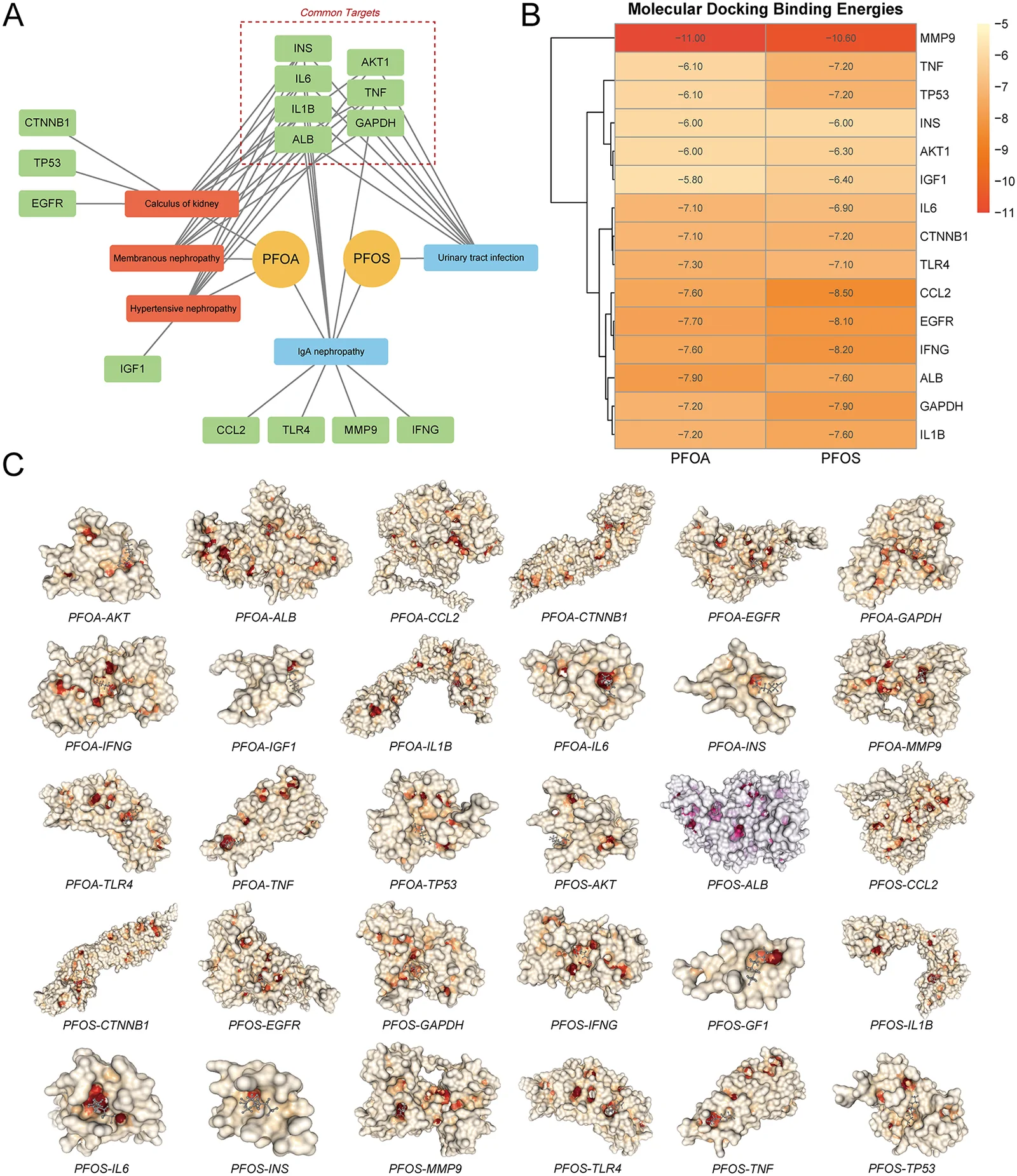
Candidate network construction and molecular docking simulation. **(A)** Association network and candidate target map of PFAS-related kidney disease outcomes. Red boxes indicate kidney disease outcomes with positive MR associations with genetically predicted PFAS levels, and blue boxes indicate kidney disease outcomes with inverse MR associations. The green boxes indicate network-prioritized candidate targets. **(B)** Binding energy heat map of molecular docking. **(C)** Detailed docking results for molecular docking.

Through the above analyses, we constructed an integrated candidate network linking genetically predicted PFAS-related traits, kidney disease outcomes, network-topological hub targets, and MGI phenotype annotations, as shown in Fig 10A. The map includes two PFAS-related traits (PFOA and PFOS), five kidney disease outcomes, and their positive or inverse MR associations. A total of 15 topological nodes were identified from the integrated PPI analyses. Among them, several targets appeared as network-prioritized candidates in specific PFAS–kidney disease outcome networks, including CTNNB1, TP53, and EGFR for PFOA–calculus of kidney, IGF1 for PFOA–hypertensive nephropathy, and CCL2, TLR4, MMP9, and IFNG for PFOA/PFOS–IgA nephropathy. In contrast, IL6, TNF, and IL1B were repeatedly identified across nephropathy-related networks and were therefore interpreted as shared inflammatory response nodes rather than association-specific candidate targets. Ubiquitous or highly abundant proteins, such as GAPDH and ALB, were retained as algorithmically identified network nodes but were not emphasized as disease-specific candidate targets.

Subsequently, molecular docking simulations were conducted for these 15 candidate proteins and two PFAS compounds to estimate their predicted binding compatibility. The docking analysis predicted favorable binding energies between PFOA/PFOS and several selected proteins, with MMP9 showing the lowest predicted binding energies for both PFAS (Fig 10B). These results provided an in silico binding-prioritization layer for selecting candidate proteins for future experimental follow-up. Fig 10C presents detailed docking results for the hub targets with PFOA and PFOS.

To further evaluate whether PFAS showed preferential predicted binding relative to non-PFAS ligands, we performed additional control docking analyses for the eight association-enriched candidate targets, including CCL2, CTNNB1, EGFR, IFNG, IGF1, MMP9, TLR4, and TP53. Glycine and butyric acid were selected as representative non-PFAS negative-control ligands, while octanoic acid was included as a non-fluorinated C8 carboxylic acid comparator for PFOA. Across these eight candidate targets, PFOA and PFOS showed more favorable predicted binding energies than the selected control ligands. The predicted binding energies ranged from −11.0 to −5.8 kcal/mol for PFOA and from −10.6 to −6.4 kcal/mol for PFOS, whereas those of the control ligands ranged from −5.9 to −2.3 kcal/mol. The full control docking results are provided in S1 Fig. These findings support the use of docking as an in silico prioritization step and require experimental evaluation in protein-binding or kidney-relevant exposure models.

## 4. Discussion

Our study integrated Mendelian randomization and computational toxicology to examine the associations between genetically predicted circulating PFAS levels and multiple kidney diseases. Genetically predicted higher PFOA levels were inversely associated with IgA nephropathy, but positively associated with hypertensive nephropathy and calculus of kidney. A primary association was also observed between PFOA and membranous nephropathy, although this association was not retained after targeted SNP-exclusion analyses. Genetically predicted higher PFOS levels were inversely associated with urinary tract infection and IgA nephropathy. Based on these genetic epidemiological findings, we constructed an integrated candidate toxicogenomic network linking PFOA/PFOS, kidney disease outcomes, and candidate molecular targets. Several targets appeared as network-prioritized candidates in specific PFAS–kidney disease outcome networks, including CTNNB1, TP53, and EGFR for PFOA–calculus of kidney, IGF1 for PFOA–hypertensive nephropathy, and CCL2, TLR4, MMP9, and IFNG for PFOA/PFOS–IgA nephropathy. In contrast, IL6, TNF, and IL1B were repeatedly identified across nephropathy-related networks and were therefore interpreted as shared inflammatory response nodes rather than association-specific candidate targets. Molecular docking added an in silico binding-prioritization layer for selected candidate proteins. Overall, these findings provide a hypothesis-generating framework in which PFAS-related genetic associations were accompanied by enrichment of immune- and inflammation-related candidate pathway themes. Broadly connected inflammatory mediators and ubiquitous or highly abundant proteins, such as IL6, TNF, IL1B, GAPDH, and ALB, should therefore be interpreted as shared network-context nodes rather than disease-selective candidate targets.

Previous epidemiological studies have generally linked PFAS exposure to adverse renal outcomes, although the direction and magnitude of associations vary across populations, PFAS congeners, and renal endpoints. Cross-sectional and longitudinal studies have reported associations between serum PFAS concentrations and altered kidney function, including lower eGFR, increased risk of reduced renal function, or renal function decline in susceptible populations [5,39]. Studies in CKD patients and population-based cohorts further suggest that PFAS mixtures may be associated with renal function deterioration [40,41], while recent studies have also linked PFAS exposure to kidney stone risk through inflammatory and metabolic pathways [9]. However, observational findings are difficult to interpret because serum PFAS concentrations reflect not only external exposure, but also protein binding, tissue distribution, renal filtration, and tubular transport [42–44]. Reduced kidney function may also decrease PFAS elimination and thereby increase circulating PFAS levels, raising the possibility of reverse causation in conventional epidemiological studies [45]. Therefore, our MR analysis should be viewed as complementary to, rather than a replacement for, observational evidence. Importantly, the MR estimates in the present study reflect genetically predicted circulating PFOA and PFOS levels, not directly measured environmental exposure levels. These genetic proxies may capture inherited differences in PFAS handling, distribution, binding, or clearance, rather than exposure dose itself.

It is important to distinguish exposure-associated instrumental loci from the candidate molecular targets identified in the computational toxicology analysis. The lead instrumental variants, particularly rs35008345 in SLC22A11 and rs141471965 in an ABCG2-related locus, suggest that genetically predicted circulating PFAS levels may partly reflect inherited differences in transporter-mediated PFAS handling, distribution, or elimination [35]. This interpretation is consistent with the known roles of OAT4 and ABCG2 in renal organic anion or xenobiotic transport [34,36]. However, direct functional evidence linking most individual SNPs to PFAS toxicokinetics remains limited. Therefore, these variants should not be interpreted as candidate kidney disease genes, but rather as genetic proxies for circulating PFAS levels.

The inverse association between genetically predicted PFOA/PFOS levels and IgA nephropathy requires cautious interpretation. One possible biological explanation is that genetically predicted PFAS-related variation may be linked to immune regulation in a disease-context-dependent manner. Because IgA nephropathy is characterized by mucosal immune dysregulation, aberrant IgA1 production, immune-complex deposition, complement activation, and downstream glomerular inflammation [46–48], genetically predicted PFAS-related variation in immune signaling could theoretically influence IgA nephropathy risk in a direction different from that observed for hypertensive nephropathy or kidney calculus [49]. However, this explanation remains speculative and should not be interpreted as evidence that PFAS exposure is protective against IgA nephropathy. Alternative explanations should also be considered. First, the genetic instruments may reflect interindividual differences in PFAS metabolism, plasma protein binding, tissue distribution, or renal clearance rather than external PFAS exposure itself. Second, the use of a suggestive genome-wide threshold may increase instrument availability but may also reduce exposure specificity. Third, although sensitivity analyses did not show strong evidence of pleiotropy, residual horizontal pleiotropy, limited statistical power, and phenotype heterogeneity cannot be fully excluded. Therefore, the inverse association observed for IgA nephropathy should be considered hypothesis-generating and requires validation in independent genetic datasets and PFAS-exposed populations.

CCL2, TLR4, MMP9, and IFNG appeared as network-prioritized candidate targets in the PFAS–IgA nephropathy network. CCL2 (MCP‑1) encodes a chemokine that recruits monocytes/macrophages. Urinary exosomal CCL2 mRNA is markedly elevated in IgA nephropathy patients and correlates with worse estimated glomerular filtration rate and tubulo‑interstitial inflammation; high exosomal CCL2 at biopsy predicts future renal function decline [50]. The Toll‑like receptor TLR4, important for mucosal immunity, is highly expressed in renal tissue of IgA nephropathy patients; expression of TLR4 in peripheral blood mononuclear cells correlates with haematuria and proteinuria, and TLR4 signalling has been reported to induce pro‑inflammatory mediators that damage mesangial cells [51]. Matrix metalloproteinase‑9 (MMP‑9) is up‑regulated in several glomerular diseases; increased glomerular MMP‑9 has been reported in IgA nephropathy and other forms of nephritis [52], supporting its role in extracellular matrix remodelling and glomerular injury. Interferon‑γ (IFNG) is a Th1 cytokine; serum IFN‑γ and urinary MCP‑1 levels are elevated in IgA nephropathy patients and correlate with proteinuria, and IFN‑γ stimulates mesangial cells to produce MCP‑1 mRNA and protein [53]. These findings suggest that CCL2–TLR4–MMP9–IFNG-related inflammatory signaling may provide a biologically plausible context for the PFAS–IgA nephropathy associations observed in this study, particularly involving macrophage recruitment, innate immune activation, and extracellular matrix remodeling.

IGF1 appeared as a network-prioritized candidate target in the PFOA–hypertensive nephropathy network. Insulin‑like growth factor‑1 (IGF‑1) is essential for renal growth and repair. Although hypertensive nephropathy–specific data are limited, IGF‑1 overexpression in diabetic kidney disease induces kidney enlargement, renal cell proliferation, mesangial expansion and inflammation [54]. An IGF‑1 receptor inhibitor alleviated these pathological changes and reduced inflammatory cytokines without affecting blood pressure [54], suggesting that excessive IGF‑1 signalling can promote renal hypertrophic and inflammatory responses in kidney disease contexts. Therefore, IGF1 may provide a plausible disease-contextual candidate target for the PFOA–hypertensive nephropathy network. Further studies are needed to evaluate whether IGF1-related renal growth, proliferative, or inflammatory responses are involved in PFAS-related hypertensive nephropathy models.

CTNNB1, TP53, and EGFR appeared as network-prioritized candidate targets in the PFOA–calculus of kidney network. CTNNB1 encodes β‑catenin, a key component of the Wnt/β‑catenin pathway. In vitro, sodium oxalate exposure (a risk factor for calcium‑oxalate stones) suppresses β‑catenin signalling and reduces epithelial barrier function; pharmacological activation of Wnt/β‑catenin with a GSK‑3β inhibitor increases β‑catenin levels and protects against oxalate‑induced epithelial injury [55]. These data suggest that β-catenin helps maintain renal epithelial integrity under oxalate-associated injury conditions, and its down-regulation may contribute to stone-related epithelial vulnerability. TP53 encodes p53, a tumour suppressor that influences ferroptosis. p53 inhibits cystine uptake and down‑regulates SLC7A11 and GPX4, lowering antioxidant capacity and promoting reactive oxygen species accumulation [56]; such oxidative-stress-related effects may contribute to crystal-associated epithelial injury and provide a plausible context for calculus formation. EGFR (epidermal growth factor receptor) regulates cell proliferation and survival. Although EGFR was prioritized in the PFOA–calculus of kidney network, most available renal evidence relates to hypertensive nephropathy: EGFR expression increases in hypertensive kidneys, and EGFR inhibitors prevent renal fibrosis and proteinuria, whereas EGFR activation promotes vasoconstriction and glomerular injury [57,58]. EGFR may therefore be considered a broader tubular injury and repair-related candidate in this network; however, direct evidence in nephrolithiasis remains limited, highlighting the need for targeted studies.

IL6, TNF, and IL1B appeared as common inflammatory nodes across multiple PFAS–kidney disease networks. Given their broad roles in inflammation and immune regulation, these genes are not readily interpretable as PFAS-specific or disease-selective targets. Rather, their repeated appearance may indicate convergence on general inflammatory and immune-response pathways across multiple PFAS–kidney disease networks. IL‑6 is secreted by mesangial cells upon stimulation with polymeric IgA1; renin‑angiotensin system blockers reduce IL‑6 production, while complement activation increases IL‑6 and TGF‑β1 release; blocking IL‑6 reduces mesangial proliferation and inflammatory cytokines [59]. These observations highlight the central role of IL‑6 in IgA nephropathy and other inflammatory nephropathies. TNF‑α is a key pro‑inflammatory cytokine; in a nephrotoxic nephritis model, deletion of T‑cell‑derived TNF‑α increases kidney injury and fibrosis, whereas TNF‑α produced by T cells attenuates injury by inhibiting Th17 responses and neutrophil infiltration [60,61]. Thus, TNF‑α has both pathogenic and protective roles depending on cellular source and disease context. IL‑1β is another pivotal cytokine; leukocyte‑derived IL‑1β promotes TNF production and glomerular injury in crescentic glomerulonephritis, while absence of leukocyte IL‑1β protects against injury [62]. Isolated glomeruli do not produce mature IL‑1β, and infiltrating immune cells are major sources [63]; inflammasome activation (caspase‑1, IL‑1β, IL‑18) correlates with albuminuria severity and albumin uptake induces IL‑1β and TNF mRNA in podocytes [63]. Together, IL-6, TNF-α, and IL-1β form a cytokine network that orchestrates inflammatory and immune responses across different kidney diseases, providing a shared inflammatory context for the PFAS–kidney disease candidate networks identified in this study.

It should also be noted that literature-supported disease relevance of these genes and predicted PFAS–protein binding represent two different levels of evidence. Many of the discussed targets, including IL6, TNF, IL1B, CCL2, MMP9, and TLR4, are known to be upregulated in inflammatory or immune-mediated kidney injury [64–68]. However, their increased expression in kidney disease should be interpreted as disease-contextual evidence supporting biological relevance, rather than as evidence that PFAS directly upregulates these genes. Similarly, docking results provide an in silico estimate of ligand–protein binding compatibility and cannot determine whether PFAS alters the expression, activity, stability, or downstream function of these targets. Therefore, whether PFAS directly regulates these targets or modifies their downstream functions remains a testable hypothesis for future experimental studies.

This study has several limitations. First, although the MR analysis suggested associations between genetically predicted circulating PFAS levels and certain kidney diseases, residual horizontal pleiotropy, limited instrument specificity, and other potential biases cannot be fully excluded. Moreover, the genetic instruments used here proxy genetically predicted circulating PFOA and PFOS levels rather than directly measured environmental exposure. Thus, the MR estimates may partly reflect inherited variation in PFAS toxicokinetics, including protein binding, tissue distribution, renal transport, and clearance, rather than effects of external PFAS exposure dose. Second, because the available PFAS metabolomics GWAS had a relatively modest sample size, instrumental SNPs were selected using a suggestive significance threshold of P < 1 × 10^−5^. Although all retained instruments had F-statistics greater than 10, this less stringent threshold may increase the possibility of including variants with weaker or less exposure-specific associations. A sensitivity analysis using the conventional genome-wide significance threshold of P < 5 × 10^−8^ was not feasible because too few independent SNPs were available after LD clumping and harmonization. Therefore, the MR findings should be interpreted cautiously and require validation when larger PFAS GWAS datasets become available. Third, most GWAS datasets used in this study were derived from individuals of European ancestry. Although this design helps reduce population stratification in two-sample MR, it may limit the generalizability of the findings to non-European populations with different genetic backgrounds, PFAS exposure profiles, environmental contexts, and kidney disease risks. Differences in outcome definitions, diagnostic criteria, sample size, and disease severity across GWAS resources may also have contributed to phenotype heterogeneity. In addition, in the targeted SNP-exclusion analyses, some retained or directionally consistent associations showed small estimated effect sizes, particularly PFOA–hypertensive nephropathy and PFOA–calculus of kidney. These findings should therefore not be overextended to imply large individual-level clinical effects or strong risk-prediction utility, and their biological relevance requires further validation.

For the computational toxicology analyses, the results should be interpreted cautiously. Target prediction databases, disease-target databases, and PPI networks are based on existing annotations and literature-derived evidence, making the prioritized candidate targets susceptible to database coverage, annotation density, and network centrality biases. PPI-based hub identification may preferentially select highly connected inflammatory mediators, such as IL6, TNF, and IL1B, which may reflect shared inflammatory responses rather than association-specific mechanisms. Likewise, ubiquitous or highly abundant proteins, such as GAPDH and ALB, may be overrepresented because of broad biological connectivity and were therefore not emphasized as disease-specific or PFAS-specific mechanistic targets. Molecular docking provides in silico estimates of ligand–protein binding compatibility. These results do not demonstrate direct PFAS–protein binding under physiological conditions, nor do they prove functional modulation, pathway activation, or causal involvement in kidney disease. Because this study did not include transcriptomic, proteomic, or functional assays, it cannot determine whether PFAS exposure alters the expression, abundance, stability, or activity of the predicted targets. Therefore, the docking results should be viewed as an in silico prioritization layer for candidate selection rather than as validation of molecular mechanisms or direct PFAS-mediated gene regulation. Future studies using larger PFAS GWAS datasets with diverse ancestral backgrounds, kidney-relevant cell types, tissue-specific expression data, PFAS exposure models, and functional experiments are needed to validate the biological relevance of the candidate targets and pathway themes. Despite these limitations, our integrative approach provides a hypothesis-generating framework for prioritizing candidate biological processes, including immune activation, oxidative stress, cell-death regulation, and epithelial barrier dysfunction, that merit further experimental investigation.

## 5. Conclusion

This study integrated genetic epidemiology and computational toxicology to examine the associations between genetically predicted circulating PFAS levels and kidney disease outcomes. Our findings identified several PFAS–kidney disease associations with different levels of robustness across sensitivity analyses, including PFOA–IgA nephropathy, PFOS–IgA nephropathy, PFOA–hypertensive nephropathy, PFOA–calculus of kidney, and PFOS–urinary tract infection. The primary PFOA–membranous nephropathy association was not retained after targeted SNP-exclusion analyses and should therefore be interpreted cautiously rather than as an established causal association. Computational toxicology analyses further prioritized database-derived candidate molecular targets and pathway themes that may provide biological context for these associations, particularly those related to inflammation, immune response, oxidative stress, and apoptosis. These findings should be interpreted as hypothesis-generating and require further validation in kidney-relevant experimental models. Overall, this study provides an integrative framework for exploring potential links among genetically predicted PFAS-related traits, kidney disease outcomes, and candidate toxicogenomic pathway themes.

## Supporting information

S1 Table Detailed SNP-level information for circulating PFAS levels. (XLSX)

S2 Table Targets for PFOA and PFOS. (XLSX)

S3 Table Targets for IgA nephropathy. (XLSX)

S4 Table Targets for membranous nephropathy. (XLSX)

S5 Table Targets for hypertensive nephropathy. (XLSX)

S6 Table Targets for calculus of kidney. (XLSX)

S7 Table Targets for urinary tract infection. (XLSX)

S8 Table SNP-to-gene annotation of PFAS genetic instruments. (XLSX)

S9 Table Functional annotation and kidney-related evidence stratification of genes mapped to PFAS instrumental SNPs. (XLSX)

S10 Table Kidney-related OpenGWAS/PheWAS evidence for PFAS instrumental SNPs. (XLSX)

S11 Table Targeted SNP-exclusion MR sensitivity analyses. (XLSX)

S12 Table Heterogeneity diagnostics for targeted SNP-exclusion MR analyses. (XLSX)

S13 Table Directional pleiotropy diagnostics for targeted SNP-exclusion MR analyses. (XLSX)

S14 Table Leave-one-out diagnostics after targeted SNP exclusion. (XLSX)

S15 Table Literature-supported renal/urinary and functional phenotype context for network-prioritized candidate targets. (XLSX)

S1 Fig Predicted binding energies of control ligands with eight association-enriched candidate targets. (A) Heatmap showing the predicted binding energies of glycine, butyric acid, and octanoic acid with eight association-enriched candidate targets, including CCL2, CTNNB1, EGFR, IFNG, IGF1, MMP9, TLR4, and TP53. Glycine and butyric acid were selected as representative non-PFAS control ligands, whereas octanoic acid was included as a non-fluorinated C8 carboxylic acid comparator for PFOA. Docking was performed using the same CB-Dock2 workflow as used for PFOA and PFOS. Binding energies are shown in kcal/mol. (B) Detailed docking results of the control ligands with the eight association-enriched candidate targets.
(TIF)

S1 Text SNP Annotation, Kidney-related PheWAS Screening, and Targeted SNP-exclusion MR Sensitivity Analyses. (DOCX)
