# Supplementary material for: Per- and polyfluoroalkyl substances and kidney disease: Genetic associations and computational prioritization of candidate toxicogenomic pathways
Source: PLoS Comput Biol. 2026 Sep 17;22(9):e1014665. doi: 10.1371/journal.pcbi.1014665 (PMC13596822; doi:10.1371/journal.pcbi.1014665)
Supplement: Supplementary file 1 [file pcbi.1014665.s017.docx]

**Supplementary Text**

**Supplementary Note: SNP Annotation, Kidney-related PheWAS Screening, and Targeted SNP-exclusion MR Sensitivity Analyses**

***1. SNP-to-gene annotation of PFAS genetic instruments***

To clarify the biological relevance of genetic variants used as instruments for PFAS exposure, we conducted comprehensive annotation of all independent SNPs (*P* < 1×10⁻⁵) employed in the MR analysis. Each SNP was mapped to its nearest gene or known expression quantitative trait loci (eQTL) target using Ensembl (v109, https://www.ensembl.org) and GTEx databases (v8, https://gtexportal.org) (if available). This mapping yielded 55 unique protein-coding genes, listed below:

| **id** | **chr** | **start** | **end** | **strand** | **gene_ids** | **gene_names** | **3_prime_utr_variant** | **nmd_transcript_variant** | **intron_variant** | **missense_variant** | **stop_gained** |
| --- | --- | --- | --- | --- | --- | --- | --- | --- | --- | --- | --- |
| rs141471965 | 4 | 88125050 | 88125050 | + | ENSG00000118777 | *ABCG2* | 0 | 0 | 4 | 0 | 0 |
| rs9809948 | 3 | 38459156 | 38459156 | + | ENSG00000114739 | *ACVR2B* | 0 | 0 | 3 | 0 | 0 |
| rs2306053 | 5 | 7784583 | 7784583 | + | ENSG00000078295 | *ADCY2* | 0 | 0 | 3 | 0 | 0 |
| rs143551944 | 6 | 109687045 | 109687045 | + | ENSG00000155085 | *AK9* | 0 | 0 | 2 | 0 | 0 |
| rs147707132 | 4 | 113116571 | 113116571 | + | ENSG00000145362 | *ANK2* | 0 | 0 | 13 | 0 | 0 |
| rs78352525 | 2 | 240514986 | 240514986 | + | ENSG00000144504 | *ANKMY1* | 0 | 0 | 6 | 0 | 0 |
| rs186404391 | 11 | 129042506 | 129042506 | + | ENSG00000134909 | *ARHGAP32* | 0 | 0 | 3 | 0 | 0 |
| rs9614577 | 22 | 44840735 | 44840735 | + | ENSG00000241484 | *ARHGAP8* | 0 | 0 | 4 | 0 | 0 |
| rs6935886 | 6 | 156928844 | 156928844 | + | ENSG00000049618 | *ARID1B* | 0 | 0 | 4 | 0 | 0 |
| rs142317920 | 15 | 80543842 | 80543842 | + | ENSG00000172379 | *ARNT2* | 0 | 0 | 2 | 0 | 0 |
| rs396050 | 6 | 25479571 | 25479571 | + | ENSG00000079691 | *CARMIL1* | 0 | 0 | 6 | 0 | 0 |
| rs117568662 | 16 | 65100809 | 65100809 | + | ENSG00000140937 | *CDH11* | 0 | 0 | 1 | 0 | 0 |
| rs72767818 | 9 | 132533247 | 132533247 | + | ENSG00000188523 | *CFAP77* | 0 | 0 | 1 | 0 | 0 |
| rs62208501 | 20 | 41500916 | 41500916 | + | ENSG00000124177 | *CHD6* | 0 | 0 | 4 | 0 | 0 |
| rs145825225 | 8 | 113055569 | 113055569 | + | ENSG00000164796 | *CSMD3* | 0 | 0 | 4 | 0 | 0 |
| rs10099460 | 8 | 1045786 | 1045786 | + | ENSG00000198010 | *DLGAP2* | 0 | 0 | 2 | 0 | 0 |
| rs187310156 | 2 | 84766861 | 84766861 | + | ENSG00000115423 | *DNAH6* | 0 | 0 | 1 | 0 | 0 |
| rs2369463 | 12 | 77038743 | 77038743 | + | ENSG00000165891 | *E2F7* | 0 | 0 | 4 | 0 | 0 |
| rs114686372 | 2 | 212506784 | 212506784 | + | ENSG00000178568 | *ERBB4* | 0 | 0 | 3 | 0 | 0 |
| rs74464512 | 16 | 74723227 | 74723227 | + | ENSG00000103089 | *FA2H* | 0 | 0 | 1 | 0 | 0 |
| rs114844958 | 10 | 14150063 | 14150063 | + | ENSG00000151474 | *FRMD4A* | 0 | 0 | 2 | 0 | 0 |
| rs3795737 | 1 | 156727264 | 156727264 | + | ENSG00000143319 | *ISG20L2* | 0 | 0 | 0 | 4 | 0 |
| rs138620492 | 10 | 77316362 | 77316362 | + | ENSG00000156113 | *KCNMA1* | 0 | 0 | 15 | 0 | 0 |
| rs71439202 | 2 | 25942865 | 25942865 | + | ENSG00000084731 | *KIF3C* | 0 | 0 | 2 | 0 | 0 |
| rs73164873 | 22 | 21051257 | 21051257 | + | ENSG00000187905 | *LRRC74B* | 0 | 0 | 1 | 0 | 0 |
| rs59494605 | 6 | 43638011 | 43638011 | + | ENSG00000124688 | *MAD2L1BP* | 0 | 0 | 2 | 0 | 0 |
| rs7788282 | 7 | 78051952 | 78051952 | + | ENSG00000187391 | *MAGI2* | 0 | 0 | 4 | 0 | 0 |
| rs57965817 | 12 | 49563320 | 49563320 | + | ENSG00000187778 | *MCRS1* | 0 | 0 | 2 | 0 | 0 |
| rs140776996 | 14 | 64428834 | 64428834 | + | ENSG00000100714 | *MTHFD1* | 0 | 0 | 1 | 0 | 0 |
| rs143450669 | 19 | 50277121 | 50277121 | + | ENSG00000105357 | *MYH14* | 0 | 0 | 5 | 0 | 0 |
| rs3822433 | 5 | 6600751 | 6600751 | + | ENSG00000037474 | *NSUN2* | 0 | 0 | 4 | 0 | 0 |
| rs184629627 | 7 | 8467436 | 8467436 | + | ENSG00000122584 | *NXPH1* | 0 | 0 | 1 | 0 | 0 |
| rs188743649 | 2 | 205306028 | 205306028 | + | ENSG00000116117 | *PARD3B* | 0 | 0 | 2 | 0 | 0 |
| rs117814140 | 18 | 80242969 | 80242969 | + | ENSG00000178184 | *PARD6G* | 0 | 0 | 2 | 0 | 0 |
| rs117098421 | 8 | 17602473 | 17602473 | + | ENSG00000104213 | *PDGFRL* | 0 | 0 | 2 | 0 | 0 |
| rs191399201 | 18 | 11077630 | 11077630 | + | ENSG00000154864 | *PIEZO2* | 0 | 0 | 9 | 0 | 0 |
| rs374054065 | 22 | 46165066 | 46165066 | + | ENSG00000186951 | *PPARA* | 0 | 0 | 2 | 0 | 0 |
| rs182382599 | 8 | 18806195 | 18806195 | + | ENSG00000156011 | *PSD3* | 0 | 0 | 2 | 0 | 0 |
| rs11571396 | 12 | 932937 | 932937 | + | ENSG00000002016 | *RAD52* | 0 | 0 | 5 | 0 | 0 |
| rs3025772 | 14 | 73121705 | 73121705 | + | ENSG00000119707 | *RBM25* | 1 | 0 | 0 | 0 | 0 |
| rs535404541 | 10 | 16750282 | 16750282 | + | ENSG00000148484 | *RSU1* | 0 | 0 | 3 | 0 | 0 |
| rs147218384 | 2 | 166418920 | 166418920 | + | ENSG00000136546 | *SCN7A* | 0 | 0 | 2 | 0 | 0 |
| rs35008345 | 11 | 64556141 | 64556141 | + | ENSG00000168065 | *SLC22A11* | 0 | 0 | 0 | 0 | 1 |
| rs9420708 | 10 | 97158385 | 97158385 | + | ENSG00000187122 | *SLIT1* | 0 | 2 | 6 | 0 | 0 |
| rs72826541 | 5 | 168949668 | 168949668 | + | ENSG00000184347 | *SLIT3* | 0 | 0 | 2 | 0 | 0 |
| rs74811952 | 8 | 50555771 | 50555771 | + | ENSG00000147481 | *SNTG1* | 0 | 0 | 3 | 0 | 0 |
| rs141922951 | 4 | 7645052 | 7645052 | + | ENSG00000184985 | *SORCS2* | 0 | 0 | 3 | 0 | 0 |
| rs34427182 | 1 | 76569409 | 76569409 | + | ENSG00000184005 | *ST6GALNAC3* | 0 | 0 | 1 | 0 | 0 |
| rs7811269 | 7 | 11377733 | 11377733 | + | ENSG00000005108 | *THSD7A* | 0 | 0 | 2 | 0 | 0 |
| rs147987488 | 12 | 129619679 | 129619679 | + | ENSG00000151952 | *TMEM132D* | 0 | 0 | 1 | 0 | 0 |
| rs182132379 | 3 | 171256489 | 171256489 | + | ENSG00000154310 | *TNIK* | 0 | 0 | 3 | 0 | 0 |
| rs563296440 | 10 | 17144165 | 17144165 | + | ENSG00000107614 | *TRDMT1* | 1 | 0 | 0 | 0 | 0 |
| rs569187847 | 11 | 78213337 | 78213337 | + | ENSG00000118369 | *USP35* | 0 | 0 | 3 | 0 | 0 |
| rs192152691 | 5 | 83495982 | 83495982 | + | ENSG00000038427 | *VCAN* | 0 | 0 | 4 | 0 | 0 |
| rs189068579 | 8 | 55329325 | 55329325 | + | ENSG00000206579 | *XKR4* | 0 | 0 | 1 | 0 | 0 |

Among the mapped genes, two transporter-related loci were particularly relevant to the interpretation of PFAS genetic instruments. SLC22A11 encodes organic anion transporter 4 (OAT4), which is expressed in renal proximal tubules and participates in the renal handling of endogenous metabolites and xenobiotic organic anions. Genetic variation in SLC22A11 has also been linked to urate-related traits and renal underexcretion-type gout [1-3]. ABCG2 encodes an ATP-binding cassette efflux transporter involved in the disposition of urate, xenobiotics, and endogenous metabolites, and genetic variation in ABCG2 has been associated with serum urate levels and gout [4-6].

These annotations indicated that some PFAS-associated instruments may capture inherited variation in transporter-mediated toxicokinetics, including renal handling, tissue distribution, or clearance. However, gene-level annotation alone was not used as an automatic exclusion criterion. Nearest-gene or eQTL-based annotation does not establish that a variant influences kidney disease independently of circulating PFAS levels. Moreover, transporter-related loci may represent genuine determinants of circulating PFAS levels rather than invalid instruments. Therefore, SNP-to-gene annotation was used to characterize the biological context of the instruments and to guide targeted sensitivity analyses, but not to exclude all variants near kidney-related or transporter-related genes.

Accordingly, we applied a targeted SNP-exclusion framework in subsequent sensitivity analyses. SNPs were flagged for exclusion based on either kidney-related or kidney-adjacent OpenGWAS/PheWAS associations, or their location at a priori transporter-related lead loci involving SLC22A11 or ABCG2.

***2. Kidney-related OpenGWAS/PheWAS screening and phenotype-based SNP exclusion***

To identify variants with independent kidney-related phenotype associations, we queried the OpenGWAS database (https://gwas.mrcieu.ac.uk/) for each independent PFAS-associated instrumental SNP. Genome-wide significant SNP–trait associations were retained using a threshold of P < 5 × 10⁻⁸. Trait names and descriptions were then screened for kidney-related or kidney-adjacent phenotypes, followed by manual review of relevant hits.

Kidney-related or kidney-adjacent traits included renal function biomarkers, serum creatinine, cystatin C, estimated glomerular filtration rate, urate or serum uric acid, gout, urolithiasis or kidney stone-related traits, blood pressure, pulse pressure, and related renal or metabolic phenotypes. SNPs with genome-wide significant associations with these traits were flagged for targeted exclusion in sensitivity analyses. The OpenGWAS/PheWAS screening results, phenotype categories, and exclusion status are summarized below:

| **SNP** | **Trait** | **GWAS ID** | **EA** | **NEA** | **EAF** | **Beta** | **P** |
| --- | --- | --- | --- | --- | --- | --- | --- |
| rs141471965 | Serum urate/uric acid levels | ebi-a-GCST90018977 | T | C | 0.1145 | 1.7120E-01 | 0 |
|  | Serum urate/uric acid levels | ukb-d-30880_irnt | T | C | 0.1145 | 1.7118E-01 | 0 |
|  | Serum urate/uric acid levels | ukb-d-30880_raw | T | C | 0.1145 | 1.4147E+01 | 0 |
|  | Gout | ebi-a-GCST90038687 | T | C | 0.1123 | 1.3179E-02 | 3.304E-268 |
|  | Gout | ukb-b-13251 | T | C | 0.1133 | 1.2995E-02 | 6.095E-251 |
|  | Allopurinol use | ukb-b-15895 | T | C | 0.1133 | 1.0713E-02 | 5.395E-212 |
|  | Serum urate/uric acid levels | ieu-b-5137 | T | C | 0.1134 | 1.6673E-01 | 1.000E-200 |
|  | Gout | ukb-a-107 | T | C | 0.1136 | 1.3541E-02 | 1.600E-197 |
|  | Allopurinol use | ukb-a-142 | T | C | 0.1136 | 1.1325E-02 | 3.715E-173 |
|  | Serum urate/uric acid levels | bbj-a-57 | T | C | 0.2955 | 1.1360E-01 | 4.508E-132 |
|  | Gout | finn-b-GOUT | T | C | 0.0749 | 6.9850E-01 | 2.728E-43 |
|  | Gout | finn-b-M13_GOUT | T | C | 0.0752 | 6.9800E-01 | 9.665E-43 |
|  | Gout | finn-b-GOUT_STRICT | T | C | 0.0746 | 8.4820E-01 | 6.537E-31 |
|  | Gout | finn-b-GOUT_NOS | T | C | 0.0744 | 7.5790E-01 | 1.511E-29 |
|  | Serum urate/uric acid levels | ukb-e-30880_CSA | T | C | 0.0898 | -2.02E-01 | 4.819E-18 |
|  | Serum urate/uric acid levels | ukb-e-30880_EAS | T | C | 0.304 | -1.70E-01 | 4.610E-12 |
|  | Gout | finn-b-GOUT_IDIO | T | C | 0.0741 | 6.5900E-01 | 1.219E-10 |
|  | Urolithiasis | bbj-a-155 | T | C | 0.2946 | 1.1072E-01 | 2.617E-08 |
| rs35008345 | Serum urate/uric acid levels | ebi-a-GCST90025965 | T | C | 0.0051 | 1.4253E-01 | 5.000E-26 |
|  | Serum urate/uric acid levels | ieu-b-5137 | T | C | 0.0052 | 1.1527E-01 | 3.800E-22 |
|  | Serum urate/uric acid levels | ebi-a-GCST90014015 | T | C |  | 1.1442E-01 | 1.009E-19 |
|  | Serum urate/uric acid levels | ebi-a-GCST90018977 | T | C | 0.0053 | 1.1770E-01 | 4.807E-17 |
|  | Serum urate/uric acid levels | ukb-d-30880_irnt | T | C | 0.0053 | 1.1768E-01 | 4.834E-17 |
|  | Serum urate/uric acid levels | ukb-d-30880_raw | T | C | 0.0053 | 9.3974E+00 | 1.009E-16 |
|  | Diastolic blood pressure | ieu-b-39 | C | T | 0.7277 | 1.5130E-01 | 9.672E-15 |
|  | Serum urate/uric acid levels | ieu-b-5137 | C | T | 0.7261 | -1.20E-02 | 5.600E-10 |
|  | Serum urate/uric acid levels | ukb-d-30880_raw | C | T | 0.7235 | -1.08E+00 | 4.830E-09 |
|  | Serum urate/uric acid levels | ebi-a-GCST90018977 | C | T | 0.7317 | -1.15E-02 | 7.621E-09 |
|  | Serum urate/uric acid levels | ukb-d-30880_irnt | C | T | 0.7235 | -1.30E-02 | 1.081E-08 |
|  | Serum urate/uric acid levels | ebi-a-GCST90014015 | C | T |  | -1.11E-02 | 3.003E-08 |
| rs45499402 | Serum urate/uric acid levels | ebi-a-GCST90018977 | C | G | 0.1638 | 1.4620E-01 | 0 |
|  | Serum urate/uric acid levels | ukb-d-30880_irnt | C | G | 0.1145 | 1.7118E-01 | 0 |
|  | Serum urate/uric acid levels | ukb-d-30880_raw | C | G | 0.1145 | 1.4148E+01 | 0 |
|  | Gout | ebi-a-GCST90038687 | C | G | 0.1123 | 1.3177E-02 | 4.603E-268 |
|  | Gout | ukb-b-13251 | C | G | 0.1133 | 1.2994E-02 | 7.194E-251 |
|  | Allopurinol use | ukb-b-15895 | C | G | 0.1133 | 1.0713E-02 | 5.902E-212 |
|  | Serum urate/uric acid levels | ieu-b-5137 | C | G | 0.1134 | 1.6673E-01 | 1.000E-200 |
|  | Gout | ukb-a-107 | C | G | 0.1136 | 1.3541E-02 | 1.762E-197 |
|  | Serum urate/uric acid levels | ebi-a-GCST90018757 | C | G | 0.2947 | 1.0982E-01 | 1.400E-180 |
|  | Allopurinol use | ukb-a-142 | C | G | 0.1136 | 1.1325E-02 | 4.083E-173 |
|  | Serum urate/uric acid levels | bbj-a-57 | C | G | 0.2956 | 1.1360E-01 | 4.753E-132 |
|  | Gout | finn-b-GOUT | C | G | 0.0748 | 7.0100E-01 | 1.435E-43 |
|  | Gout | finn-b-M13_GOUT | C | G | 0.0751 | 7.0050E-01 | 5.030E-43 |
|  | Gout | finn-b-GOUT_STRICT | C | G | 0.0745 | 8.4950E-01 | 5.400E-31 |
|  | Gout | finn-b-GOUT_NOS | C | G | 0.0743 | 7.6180E-01 | 8.183E-30 |
|  | Serum urate/uric acid levels | ukb-e-30880_CSA | C | G | 0.0898 | -2.02E-01 | 5.631E-18 |
|  | Serum urate/uric acid levels | ukb-e-30880_EAS | C | G | 0.304 | -1.70E-01 | 4.844E-12 |
|  | Urolithiasis | ebi-a-GCST90018935 | C | G | 0.1543 | 9.6100E-02 | 1.108E-11 |
|  | Gout | finn-b-GOUT_IDIO | C | G | 0.074 | 6.6060E-01 | 1.113E-10 |
|  | Urolithiasis | ebi-a-GCST90018715 | C | G | 0.2939 | 9.5836E-02 | 1.466E-09 |
|  | Urolithiasis | bbj-a-155 | C | G | 0.2946 | 1.1012E-01 | 3.351E-08 |
| rs57965817 | Pulse pressure | ieu-b-5140 | G | A | 0.129 | -2.17E-01 | 1.300E-08 |
| rs71439202 | Cystatin C | ebi-a-GCST90014003 | C | T |  | 3.3531E-02 | 6.819E-10 |
|  | Serum urate/uric acid levels | ieu-b-5137 | C | T | 0.0326 | 2.9163E-02 | 1.600E-09 |
|  | Serum urate/uric acid levels | ebi-a-GCST90014015 | C | T |  | 3.0959E-02 | 1.746E-09 |
|  | Creatinine | ebi-a-GCST90014001 | C | T |  | 2.8322E-02 | 1.462E-08 |
|  | Cystatin C | ukb-d-30720_irnt | C | T | 0.0321 | 3.4998E-02 | 2.708E-08 |

Using this phenotype-based screening framework, six SNPs showed kidney-related or kidney-adjacent OpenGWAS/PheWAS evidence: rs141471965, rs35008345, rs396050, rs45499402, rs57965817, and rs71439202. These variants were mainly associated with urate or serum uric acid levels, gout, urolithiasis, blood pressure, pulse pressure, cystatin C, or creatinine-related traits.

***3. Targeted SNP-exclusion MR sensitivity analyses***

Based on the SNP annotation and OpenGWAS/PheWAS screening described above, we repeated the MR analyses under three targeted SNP-exclusion scenarios.

● First, we excluded transporter-related lead variants of primary concern. For PFOA, this exclusion removed the SLC22A11-related lead SNP rs35008345. For PFOS, this exclusion removed the ABCG2/SLC22A11-related lead SNPs rs141471965 and rs76713685.

● Second, we excluded SNPs with genome-wide significant kidney-related or kidney-adjacent OpenGWAS/PheWAS associations. For PFOA, this exclusion removed rs35008345, rs45499402, and rs396050. For PFOS, this exclusion removed rs141471965, rs57965817, and rs71439202.

● Third, we performed a combined exclusion analysis in which both transporter-related lead SNPs and kidney-related OpenGWAS/PheWAS SNPs were removed. For PFOA, the combined exclusion removed rs35008345, rs45499402, and rs396050. For PFOS, the combined exclusion removed rs141471965, rs76713685, rs57965817, and rs71439202.

After removing the corresponding SNPs from the harmonized instrument sets, MR analyses were repeated using the same analytical pipeline as in the primary analysis. MR estimates were summarized on the β scale. Wald-ratio estimates were reported when only one valid instrument remained. When no valid instrument remained after exclusion, the corresponding sensitivity analysis was considered not estimable.

***4. Results of targeted SNP-exclusion MR sensitivity analyses***

After excluding SLC22A11/ABCG2-related lead variants, kidney-related OpenGWAS/PheWAS variants, or both SNP sets, several associations remained statistically significant or directionally consistent. The PFOA–IgA nephropathy association remained significant across all three exclusion scenarios, although the estimate was based on a single Wald-ratio instrument (β = -2.513, P = 0.015). The PFOA–hypertensive nephropathy association also remained significant after all exclusion strategies (β = 0.001, P < 0.001). The PFOS–urinary tract infection association remained inverse (β < 0) and statistically significant after all exclusion strategies in both SPA-corrected and Firth-corrected outcome definitions.

| **Association** | **Excluding SLC22A11/ABCG2**  **-related lead SNPs** | **Excluding kidney-related PheWAS SNPs** | **Excluding both SNP sets** | **Summary** |
| --- | --- | --- | --- | --- |
| PFOA → IgA nephropathy | β = -2.513, P = 0.015 | β = -2.513, P = 0.015 | β = -2.513, P = 0.015 | Retained |
| PFOA → membranous nephropathy | β = 0.444, P = 0.160 | β = 0.157, P = 0.292 | β = 0.157, P = 0.292 | Not retained |
| PFOA → hypertensive nephropathy | β = 0.001, P < 0.001 | β = 0.001, P < 0.001 | β = 0.001, P < 0.001 | Retained |
| PFOA → calculus of kidney | β = 0.001, P = 0.034 | β = 0.001, P = 0.059 | β = 0.001, P = 0.059 | Attenuated after PheWAS exclusion |
| PFOS → IgA nephropathy | Not estimable | Not estimable | Not estimable | No valid instrument remained after exclusion |
| PFOS → urinary tract infection, SPA | β = -0.071, P = 0.002 | β = -0.074, P = 0.002 | β = -0.074, P = 0.002 | Retained |
| PFOS → urinary tract infection, Firth | β = -0.071, P = 0.002 | β = -0.075, P = 0.002 | β = -0.075, P = 0.002 | Retained |

The PFOA–calculus of kidney association remained directionally consistent and nominally significant after excluding SLC22A11/ ABCG2-related lead variants (β = 0.001, P = 0.034), but was attenuated after excluding kidney-related OpenGWAS/PheWAS variants or both SNP sets (β = 0.001, P = 0.059). The PFOA–membranous nephropathy association was not retained after targeted SNP exclusion. The PFOS–IgA nephropathy association was not estimable under the targeted exclusion framework because no valid instrument remained after exclusion.

For retained multi-SNP analyses, Cochran’s Q tests were used to evaluate heterogeneity and MR-Egger intercept tests were used to evaluate directional pleiotropy when sufficient instruments were available. Overall, the sensitivity diagnostics did not indicate substantial heterogeneity, directional pleiotropy, or single-SNP-driven instability for the retained or directionally consistent associations after targeted SNP exclusion, including PFOA–hypertensive nephropathy, PFOA–calculus of kidney, and PFOS–urinary tract infection. For single-SNP analyses, including the PFOA–IgA nephropathy association after targeted exclusion, heterogeneity, pleiotropy, and leave-one-out diagnostics were not applicable (Tables S12-S14).

These results suggest that the robustness of the MR findings differed across kidney disease outcomes. Associations that remained significant after both transporter-locus exclusion and kidney-related OpenGWAS/PheWAS exclusion were less likely to be driven solely by the SLC22A11/ABCG2-related lead variants or by SNPs with known kidney-related phenotype associations. In contrast, attenuation of the PFOA–calculus of kidney association after excluding kidney-related OpenGWAS/PheWAS variants suggests that this association may be more sensitive to renal-handling, urate-related, or kidney-adjacent pleiotropic pathways. The loss of estimability for PFOS–IgA nephropathy reflects the limited number of valid instruments remaining after conservative exclusion rather than definitive evidence against the primary association. Overall, these targeted SNP-exclusion analyses support a cautious interpretation of genetically predicted circulating PFAS levels as exposure proxies that may partly reflect PFAS toxicokinetics, including renal transport and clearance.

***5. Functional annotation and kidney-related evidence stratification of genes mapped to PFAS instrumental SNPs***

To further characterize the biological context of genes mapped to PFAS instrumental SNPs, we expanded the SNP-to-gene annotation by summarizing the basic molecular function and kidney-related evidence for each mapped gene. Genes were stratified into renal transporter/toxicokinetic loci, genes with variant-level kidney-related OpenGWAS/PheWAS evidence, genes with reported kidney disease or renal injury context, indirect metabolic/vascular/immune-related genes, and genes with limited direct kidney evidence (Tables S9).

This annotation was used for evidence stratification rather than automatic instrument exclusion. Gene-level kidney relevance alone does not establish variant-level horizontal pleiotropy. Therefore, the functional annotation was interpreted together with OpenGWAS/PheWAS screening and targeted SNP-exclusion MR analyses. Genes such as SLC22A11 and ABCG2 were classified as high-priority transporter/toxicokinetic loci and addressed through targeted exclusion analyses. Other genes with reported kidney-related context, including NSUN2, were explicitly flagged as contextual evidence, but were not interpreted as definitive evidence of invalid instruments unless supported by variant-level kidney-related PheWAS associations.

**Reference**

1.	Zhou F, You G. Molecular insights into the structure-function relationship of organic anion transporters OATs. Pharm Res. 2007;24(1):28-36.

2.	Sakiyama M, Matsuo H, Shimizu S, Nakashima H, Nakayama A, Chiba T, et al. A common variant of organic anion transporter 4 (OAT4/SLC22A11) gene is associated with renal underexcretion type gout. Drug Metab Pharmacokinet. 2014;29(2):208-10.

3.	Yang Q, Köttgen A, Dehghan A, Smith AV, Glazer NL, Chen MH, et al. Multiple genetic loci influence serum urate levels and their relationship with gout and cardiovascular disease risk factors. Circ Cardiovasc Genet. 2010;3(6):523-30.

4.	Wakabayashi K, Tamura A, Saito H, Onishi Y, Ishikawa T. Human ABC transporter ABCG2 in xenobiotic protection and redox biology. Drug Metab Rev. 2006;38(3):371-91.

5.	Hoque KM, Dixon EE, Lewis RM, Allan J, Gamble GD, Phipps-Green AJ, et al. The ABCG2 Q141K hyperuricemia and gout associated variant illuminates the physiology of human urate excretion. Nat Commun. 2020;11(1):2767.

6.	Yamagishi K, Tanigawa T, Kitamura A, Köttgen A, Folsom AR, Iso H. The rs2231142 variant of the ABCG2 gene is associated with uric acid levels and gout among Japanese people. Rheumatology (Oxford). 2010;49(8):1461-5.
